# Comparison of on-site versus NOAA’s extreme precipitation intensity-duration-frequency estimates for six forest headwater catchments across the continental United States

**DOI:** 10.1007/s00477-023-02495-0

**Published:** 2023-10

**Authors:** Sourav Mukherjee, Devendra M. Amatya, Anna M. Jalowska, John L. Campbell, Sherri L. Johnson, Kelly Elder, Sudhanshu Panda, Johnny M. Grace, Duncan Kikoyo

**Affiliations:** 1 Center for Forested Wetlands Research, Southern Research Station, USDA Forest Service, 3734 Highway 402, Cordesville, SC 29434, USA; 2 Office of Research and Development, U.S. Environmental Protection Agency, Research Triangle Park, Durham, NC, USA; 3 Northern Research Station, USDA Forest Service, Durham, NH, USA; 4 Pacific Northwest Research Station, USDA Forest Service, Corvallis, OR, USA; 5 Rocky Mountain Research Station, USDA Forest Service, Fort Collins, CO, USA; 6 Institute of Environmental Spatial Analysis, University of North Georgia, 3820 Mundy Mill Road, Oakwood, GA 30566, USA; 7 Center for Forest Watershed Research, Southern Research Station, USDA Forest Service, 1740 S. Martin Luther King Jr. Blvd., Perry-Paige Bldg., Suite 303 North, Tallahassee, FL 32307, USA; 8 Texas A&M University, College Station, USA

**Keywords:** Regional frequency analysis, Forest service experimental forests, Headwater catchments, Design discharge, Road-stream crossing structures, NOAA Atlas 14

## Abstract

Urgency of Precipitation Intensity-Duration-Frequency (IDF) estimation using the most recent data has grown significantly due to recent intense precipitation and cloud burst circumstances impacting infrastructure caused by climate change. Given the continually available digitized up-to-date, long-term, and fine resolution precipitation dataset from the United States Department of Agriculture Forest Service’s (USDAFS) Experimental Forests and Ranges (EF) rain gauge stations, it is both important and relevant to develop precipitation IDF from onsite dataset (Onsite-IDF) that incorporates the most recent time period, aiding in the design, and planning of forest road-stream crossing structures (RSCS) in headwaters to maintain resilient forest ecosystems. Here we developed Onsite-IDFs for hourly and sub-hourly duration, and 25-yr, 50-yr, and 100-yr design return intervals (RIs) from annual maxima series (AMS) of precipitation intensities (PIs) modeled by applying Generalized Extreme Value (GEV) analysis and L-moment based parameter estimation methodology at six USDAFS EFs and compared them with precipitation IDFs obtained from the National Oceanic and Atmospheric Administration Atlas 14 (NOAA-Atlas14). A regional frequency analysis (RFA) was performed for EFs where data from multiple precipitation gauges are available. NOAA’s station-based precipitation IDFs were estimated for comparison using RFA (NOAA-RFA) at one of the EFs where NOAA-Atlas14 precipitation IDFs are unavailable. Onsite-IDFs were then evaluated against the PIs from NOAA-Atlas14 and NOAA-RFA by comparing their relative differences and storm frequencies. Results show considerable relative differences between the Onsite- and NOAA-Atlas14 (or NOAA-RFA) IDFs at these EFs, some of which are strongly dependent on the storm durations and elevation of precipitation gauges, particularly in steep, forested sites of H. J. Andrews (HJA) and Coweeta Hydrological Laboratory (CHL) EFs. At the higher elevation gauge of HJA EF, NOAA-RFA based precipitation IDFs underestimate PI of 25-yr, 50-yr, and 100-yr RIs by considerable amounts for 12-h and 24-h duration storm events relative to the Onsite-IDFs. At the low-gradient Santee (SAN) EF, the PIs of 3- to 24-h storm events with 100-yr frequency (or RI) from NOAA-Atlas14 gauges are found to be equivalent to PIs of more frequent storm events (25–50-yr RI) as estimated from the onsite dataset. Our results recommend use of the Onsite-IDF estimates for the estimation of design storm peak discharge rates at the higher elevation catchments of HJA, CHL, and SAN EF locations, particularly for longer duration events, where NOAA-based precipitation IDFs underestimate the PIs relative to the Onsite-IDFs. This underscores the importance of long-term high resolution EF data for new applications including ecological restorations and indicates that planning and design teams should use as much local data as possible or account for potential PI inconsistencies or underestimations if local data are unavailable.

## 1 Introduction

Flooding induced by extreme precipitation events has a major impact on road-stream crossing infrastructure across the United States (US), resulting in a re-evaluation of their design codes (Adeel et al. 2020; Doyle and Ketcheson 2007; Neary and Leonard 2019; Wright et al. 2019). The US Department of Agriculture Forest Service (USDAFS) is responsible for managing approximately 595,700 km of roads and thousands of road-stream crossing structures (RSCS) within the National Forest System (NFS), with the goal of providing both flood resilience and aquatic organism passage (Coffman et al. 2005; Heredia et al. 2016). Accurate estimation of design storm discharge, defined as the peak discharge for a given storm frequency, from watersheds is critical for design of RSCS and stream restoration practices. The importance of developing accurate site-specific Precipitation Intensity-Duration-Frequency (IDF) estimation with the latest data has increased substantially due to present extreme precipitation and cloud burst conditions occurring in last few decades as a consequence of climate change (Fowler et al. 2021; Westra et al. 2014). Design storm discharge can be determined by the precipitation-runoff relationship, which is dependent on several factors such as precipitation intensity, storm duration, land-use or land cover, antecedent soil-moisture conditions, soil–water storage, and geomorphology of a watershed (Berghuijs et al. 2016).

The road-stream crossing structures (RSCS), such as fords, culverts, and bridges are designed based on the hydraulic capacity of the structure to convey a specific peak discharge over an assumed life span (Donahue and Howard 1987; Rasmussen et al. 2018) to reduce the risk of flood damage and catastrophic failure. The peak discharge is calculated based on precipitation intensity (PI) of storm events of a specific duration depending on time of concentration (t_c_), and frequency (Liu et al. 2022; Martel et al. 2021; USDA 1986). In addition, PI of a specific duration and frequency is needed for mapping erosion vulnerability (Knox et al. 2015; Panda et al. 2022), which is a critical parameter for identifying the ecoregions that are most vulnerable to post-fire flash flooding, landslides, and structural failures due to sediment deposition, causing disruption to forest operations (Borga et al. 2014; Ebel et al. 2012; Thomas et al. 2021).

Precipitation IDF analysis of storm events is useful for determining the frequency of occurrence of floods that can cause structural damage to RSCS, lead to debris jams and potential erosion in road networks and headwater valleys (Jakob et al. 2020; Mamo 2015). Most of the RSCS on USDAFS land are in headwater forested catchments with small drainage areas (\ 1000 ha), usually with sub-daily t_c_ (USDA 1986; Corbin et al. 2021; Yochum et al. 2019; Walega et al. 2020). Therefore, the use of PI estimates at daily timescale for calculation of design discharge rates in small watersheds (Wright et al. 2019) may lead to underestimation of flood potential of those regions.

Rational Method (Kuichling 1889) is one of the widely used flood discharge models used in design of the RSCS in small catchments; the duration of a largest PI causing a flood of given return interval (RI) is chosen based on t_c_. Accordingly, the t_c_ for flow in such small catchments would likely be small in the sub-daily scale (Hayes and Young 2006; Zolghadr et al. 2022). Furthermore, roads and their drainage ditches on smaller watersheds can also significantly increase the magnitude of peak discharges by routing water quickly to the streams (Wemple and Jones 2003). Accordingly, PIs of a duration shorter than the daily time-scale are generally recommended to estimate design flood discharges for these watersheds smaller than 1 km^2^ (Amatya et al. 2021). However, due to a lack of long-term historical precipitation records at a fine temporal resolution (sub-hourly and sub-daily timescales), the RSCS on USDAFS lands and similar other landscapes are commonly designed using coarser daily resolution or, if available, the sub-hourly and sub-daily PIs provided by the National Oceanic and Atmospheric Administration (NOAA) Atlas 14, hereafter, referred to as ‘NOAA-Atlas14’. NOAA-Alas14 precipitation IDFs are calculated based on the regional temporal distribution of precipitation (Perica et al. 2018).

There are multiple caveats and limitations pertaining to the application of precipitation IDF from NOAA-Atlas14. Although NOAA-Atlas14 may serve as a general guideline for structural design, site specific accuracy of their published interpolated values may decline with distance from available NOAA rain gauge locations being considered (Perica et al. 2018). This is a major limitation of using NOAA-Atlas14 precipitation IDFs for design of remote, forested RSCS. Climate change induced localized rain and cloud bursts require site specific precipitation records for developing precipitation IDF to support forest RSCS design and other forest ecological management decision support systems (Rosenzweig et al. 2019). This issue is especially problematic where the spatial heterogeneity of precipitation is large, such as in complex, mountainous terrain, which is common on federal forests (Basist et al. 1994; Preece et al. 2021; Wu et al. 2020; Yu et al. 2020). In addition, irregularly spaced observation sites in the National Weather Service’s (NWS) Cooperative Observer Program (COOP) network, like many other data networks, can leave substantial data gaps in sparsely observed locations, potentially introducing error by assuming spatial homogeneity, thereby inherently ignoring local variations in topography and land use (Jalowska et al. 2021). Furthermore, sub-hourly and hourly pointwise precipitation IDFs from NOAA-Atlas14 are not available for the entire US, especially in states located in Northwestern US, including Idaho, Montana, Oregon, Washington, and Wyoming (Perica et al. 2018), where large percentages of the lands are federal forests.

Importantly, the length and recency of the data used to estimate precipitation IDFs by NOAA-Atlas14 varies among regions, and often do not account for contemporary changes in precipitation extremes, such as those documented in the Southeastern US (Bonnin et al. 2006). Most precipitation IDFs have not been updated since 2006, missing almost 15 years of some very intense precipitation events (Kunkel and Champion 2019; here authors acknowledge the September 2022 NOAA announcement of the upcoming release of updated Atlas15, planned for 2026). In some regions NOAA-Atlas14 precipitation IDFs are calculated based on data collected through 2013 and in Texas through 2017. Overall, the NOAA-Atlas14 does not include the more recent changes in precipitation extremes necessary for addressing the effect of climate change in stormwater infrastructure adaptations plans developed by EFs and other agencies. As such, in a changing climate, concepts like ‘‘100-year precipitation event’’ based on the NOAA-Atlas14 can be misleading. Particularly, PIs linked to a 100-yr RI based on the outdated NOAA-Atlas14 data may actually be linked to PIs with a frequency of less than 100-yr RI based on data that encompasses the most recent time period. USDAFS-RSCS, are commonly designed to accommodate flood flows of specific RIs or estimated using PIs obtained from the NOAA-Atlas14, but may be underestimating the risk of structural failures arising from under sizing of culverts and stream crossings (Donahue and Howard 1987). Consequently, any policy formation linked to an underestimated frequency of extreme precipitation storm events is likely to impact the monitoring-systems built for supporting the resilience of forest ecosystems, and the critical economic and environmental services they provide (Rustad et al. 2012).

Precipitation IDF curves are graphic representation of parametric, mathematical relationships between the PI, the reciprocal of non-exceedance probabilities (i.e., return periods or storm frequency), and a scale (i.e., storm duration) of temporal averaging of the precipitation intensities (which is a design value; Wanielista et al. 1996; Kout-soyiannis et al. 1998). To derive these mathematical relationships, NOAA-Atlas14 primarily uses the regional frequency analysis (RFA) methodology with L-moments (Hosking and Wallis 1997). In addition, NOAA-Atlas14 precipitation IDFs are derived from the annual maximum series (AMS; series of the largest single events that occurred in each year) of PIs, accessible from NOAA’s gauge stations. As an alternative to the NOAA’s gauge records, long-term, sub-hourly to sub-daily precipitation records are available from some of the USDAFS EFs within the NFS (Amatya and Walega 2020; Amatya et al. 2021;) for developing the precipitation IDFs. It is evident that the use of the sub-hourly and sub-daily precipitation IDFs obtained using onsite rain gauge station data in hydrological models could produce more accurate design discharge on small headwater watersheds (Amatya et al. 2021; Petroselli and Grimaldi 2018;). Despite the available alternatives to NOAA-Atlas14 precipitation IDFs, only a few studies have compared the precipitation IDFs published by NOAA to those derived from onsite precipitation records (Amatya et al. 2021; Butcher et al. 2021; Eldardiry and Habib 2020; Ombadi et al. 2018).

**Fig. 1:**
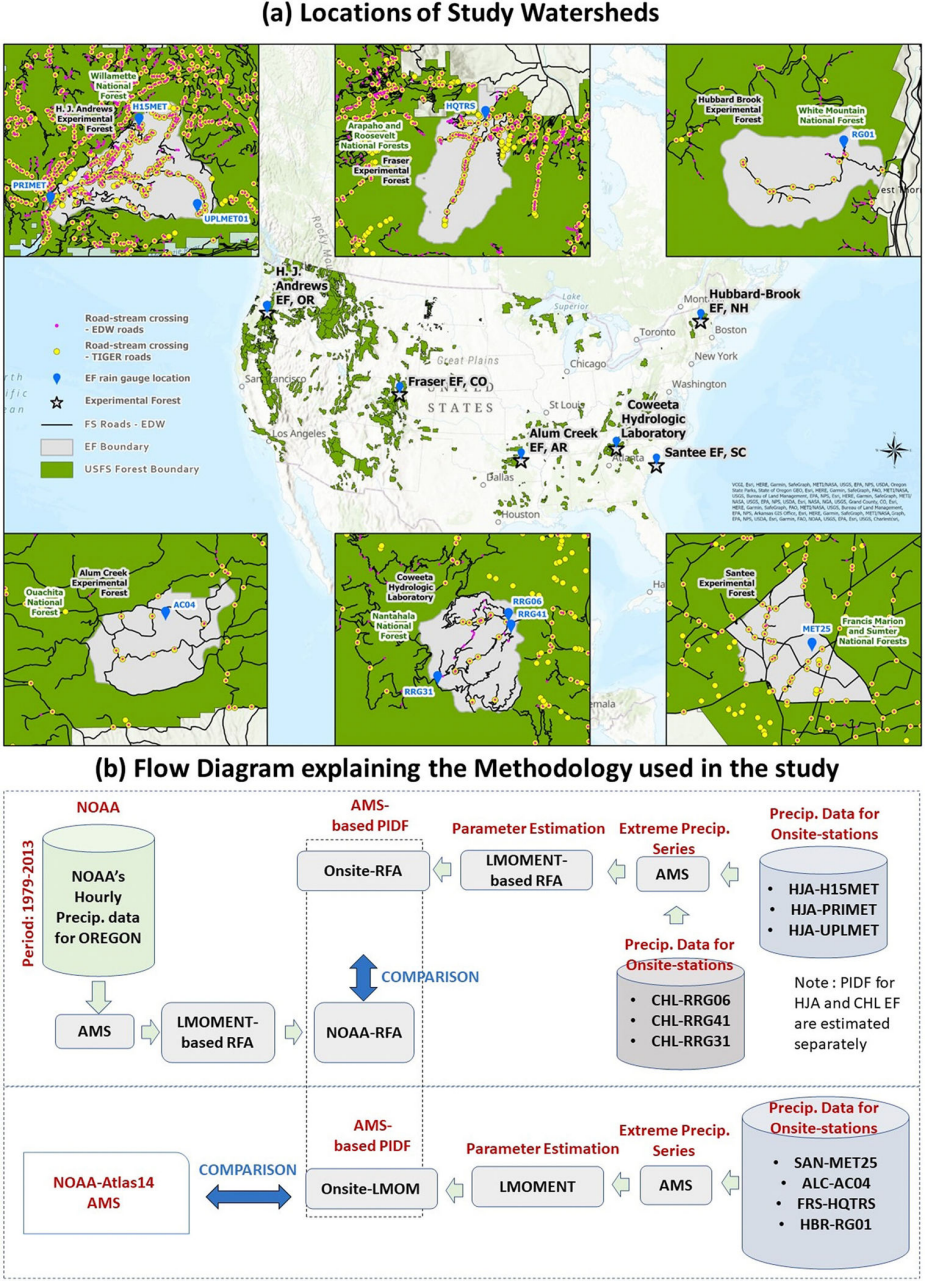
**a** Locations of the study EFs within a National Forest, major RSCS, selected onsite rain gauge stations for all six EFs included in the study, and **b** flow diagram illustrating the methodology

In this study, we conduct a comprehensive statistical analyses and estimation of onsite precipitation IDFs using ground-based observations of sub-hourly, sub-daily, and daily long-term precipitation records from rain gauges on multiple USDAFS EFs located in different climate regimes of the US, a novelty of this study, and compare these to standard NOAA-Atlas14 precipitation IDFs (Fig. 1a). The major novelty of this study rests in two areas: (1) RFA for the state of Oregon, where the HJA experimental forest is located and NOAA has not yet provided precipitation IDF estimates for the region, and (2) this is the first study of its kind to assess PIs using long-term and high resolution data for small, wooded headwater watersheds in very different eco-climatological zones across the United States. Our specific objectives include:

1. regional precipitation IDF estimation at EF locations where data from multiple onsite gauges are available and investigate the impact of varying topography on the precipitation IDFs at those locations,
2. evaluating onsite precipitation IDFs from longterm high-resolution sub-hourly, sub-daily, and daily PIs and comparing them with NOAA-Atlas14 and NOAA-RFA based precipitation IDFs, and
3. Assessing the uncertainties in design precipitation IDFs associated with estimating parameters of the extreme value distribution,

Overall, we believe that the findings of this study can help guide and inform forest land managers and design engineers who are accustomed to using the NOAA precipitation IDFs, with an alternative choice of Onsite precipitation IDF (hereafter referred to as Onsite-IDF) for application in reliable design of RSCS and ecological applications, as well as future flood risk assessment in forested watersheds within the selected EFs and beyond on ungauged watersheds in the region.

## 2 Materials and methods

### 2.1 Study sites

Onsite-IDFs are estimated using long-term fine resolution historical records of precipitation data obtained for six Experimental Forests (EFs): Horace Justin Andrews (HJA), Oregon; Santee (SAN), South Carolina; Coweeta Hydrologic Laboratory (CHL), North Carolina; Alum Creek (ALC), Arkansas; Fraser (FRS), Colorado; and Hubbard Brook (HBR), New Hampshire. The locations of the study watersheds, rain gauge stations, and major RSCS for all six EFs are illustrated in Fig. 1a. A detailed description of the study sites is provided by Amatya et al. (2021) and Harder et al. (2007) for SAN; Caldwell et al. (2016) and Laseter et al. (2012) for CHL; Adams and Loughry (2008) for AC; Likens (2013) and Campbell et al. (2021) for HBR; Johnson et al. (2021) and Fredriksen (1970) for HJA; and Alexander and Watkins (1977) for FEF. Shuttle Radar Topography Mission (SRTM) global digital elevation model (DEM) data (from https://earthexplorer.usgs.gov/), available at 1 m and 10 m (for CHL and FRS) resolution were obtained for the selected EF’s and watersheds as illustrated in Fig. S1-S6.

### 2.2 Data compilation

Long-term onsite precipitation data were compiled for the six EFs selected in the study (Fig. 1a). The EF name, rain gauge ID, time-period used in the study, finest temporal resolution, location coordinates, mean annual precipitation, and station-elevations are listed in Table 1. The finest temporal resolution and length of contiguous digital records of precipitation varied across the selected EFs and rain gauge stations. Long-term sub-hourly precipitation data were available for only two EFs, – for HJA (three rain gauge stations; station ID: HJA-H15MET, HJA-PRIMET, and HJA-UPLMET), and for HBR (one rain gauge, station ID: HBR-RG01). A previous study reported on the influence of topography on hourly to daily precipitation IDFs by analyzing data from three rain gauges at various elevations in the CHL EF region (Amatya et al. 2021). In this study, we further investigated the impact of variable topography on the (sub-hourly to daily) onsite and NOAA-based precipitation IDFs. We selected three rain-gauge stations at the HJA (Table 1; station ID: HJA-H15MET, HJA-PRI-MET, and HJA-UPLMET; Daly et al. 2019), and three rain gauges in the CHL (Table 1; station ID: CHL-RRG06, CHL-RRG41, and CHL-RRG31) which were used in Amatya et al. 2021 (Fig. 1a). Continuous digital records of hourly precipitation were available starting in 2003 for SAN (station ID: SAN-MET25), 1976 for CHL (station ID: CCHL-RRG06), 1997 for ALC (station ID: AC (04)), and 2004 for FRS (station ID: FRS-HQTRS). Following Amatya et al., (2021), missing sub-daily precipitation data for the period, 1977–1994, at the SAN-MET25 were obtained directly from the nearest Lotti gauge (also within the same Santee experimental forest), located 1.73 km southeast of and at the same elevation as MET25 station within the WS80 watershed. Similarly, missing data for the 1995–2002 period, were obtained from a NOAA station at Charleston airport. To maintain consistency in the assessment of precipitation IDFs among the EFs with the longest available records, such as SAN, CHL, and HBR, we used the same data period, 1977–2021 (45 years) for the precipitation IDF analysis (Table 1) in contrast with Amatya et al (2021) that used data through 2015 for the SAN, CHL, and ALC. For the remaining two EFs (ALC, and FRS), the full available data period was used in the analysis. Detailed procedures of onsite data collection and digitization for the CHL, Santee, and ALC are provided by Amatya et al. (2021).

NOAA Atlas-14 estimated precipitation IDFs for rain gauge locations at each of the EFs were downloaded from the Precipitation Frequency Data Server (Perica et al. 2018). However, for HJA, pointwise estimates of precipitation IDFs are not available in the NOAA Atlas-14. Therefore, we derived NOAA precipitation IDFs for this site employing Regional Frequency Analysis (RFA) using rain gauges in the Oregon with hourly precipitation data (Fig. S7), available from NOAA’s National Climatic Data Center (https://www.ncei.noaa.gov/access/metadata/landing-page/bin/iso?id=gov.noaa.ncdc:C00313). NOAA’s hourly precipitation data were obtained for the stations with the minimum data coverage (1979–2013) to match, as closely as possible, with the longest data record available for the HJA (station, HJA-PRIMET: 1979–2018). We obtained 109 such NOAA stations in Oregon. Finally, 63 NOAA stations (listed in Table S1 of Supplementary Information) were selected based on three additional criteria (Fig. S7) – (1) number of non-zero records per year should be more than 50, (2) number of years with zero precipitation should be zero, and (3) number of missing values per year should be less than 20. SRTM global digital elevation model data for the Oregon state, available from the USGS earth explorer (https://earthexplorer.usgs.gov/), was used as a covariate for spatial interpolation of NOAA PIs.

### 2.3 Regional frequency analysis (RFA) of precipitation intensities using L-moments

In this study, we performed the RFA analysis using the ‘‘*lmomRFA*’’ R-package (Hosking 2022), based on Hosking and Wallis, (1997) that reflects the same methodology as used in NOAA-Atlas14 (Perica et al. 2018). AMS of PIs was constructed for each available NOAA station by taking the highest PIs for a particular storm duration (1-, 2-, 3-, 6-, 12-, and 24-h) in each calendar year within the 1979–2013 period. The AMS of PIs is calculated by normalizing precipitation depth with the selected storm durations. Similarly, AMS for the three stations each from the HJA and CHL EF (Table 1) were included for the RFA using the onsite precipitation data. The precipitation IDFs estimated with these onsite datasets and RFA are hereafter referred to as Onsite-RFA precipitation IDFs in this study. Our approach adheres as closely as possible to the way in which the NOAA-Atlas14 precipitation IDF estimates are determined. Specifically, in most cases, precipitation IDF estimates in the NOAA-Atlas14 stem from the RFA, derived by fitting a generalized extreme value (GEV) distribution to the AMS. It should be noted that prior to estimating the precipitation IDFs, the AMS were detrended using the Sen’s slope (Cho and Jacobs 2020; Sen 1968).

The RFA using L-moments was performed with the index rainfall method, where the quantile function, $Q_{i}(F)$ of the cumulative distribution function F at a site, i (i=1,2,…N), is given as (Hosking and Wallis 1997),

(1) $$Q_{i}(F)=\mu_{i}q(F)$$

where, $\mu_{i}$ is the index-rainfall calculated as the mean of the on-site rainfall series, and q(F) is a dimensionless quantile function also known as the regional growth curve estimated with the RFA. Once the regional growth curve was obtained for a region using the L-moment statistics, the site-specific quantiles were estimated with Eq. (1). A detailed discussion of the calculation of L-moment statistics (e.g., sample mean, scale, L-kurtosis, and L-skewness) and L-moment ratios (sample L-moment coefficient of variation (L-CV)) can be found in Hosking and Wallis (1997).

The homogeneous regions were selected by performing a cluster analysis based on site characteristics for the stations selected in the RFA. We selected 109 NOAA rain gauge stations in Oregon based on data availability. The identified clusters were treated as preliminary homogeneous regions, and a heterogeneity test was performed to validate the homogeneity of the cluster using a heterogeneity measure. Preliminarily, the three USDAFS onsite gauges in each of the HJA and CHL EF were assumed to constitute a single homogenous region due to their proximity. A detailed discussion on the methodology used in the selection of homogeneous regions and the heterogeneity testing is provided in the Supplementary Material (Text S1).

To maintain consistency with the NOAA-Atlas14 methodology, we selected the GEV distribution to fit the AMS derived from the NOAA’s rain gauge records. This choice was also necessary and consistent with Amatya et al. (2021) for comparing the EF’s Onsite-IDFs with NOAA precipitation IDFs since NOAA fits GEV distribution using the AMS of PIs. To determine how well the sample data fit the GEV distribution, we performed a goodness-of-fit (GOF) test following the same procedure described in Hosking and Wallis (1997) which uses the Z-statistics. The GEV distribution was considered sufficient for fitting the AMS if it satisfied |Z |≤ 1.64 (Hosking and Wallis 1997).

Once the fit of the GEV distribution was deemed acceptable, the precipitation quantiles were then derived for each region using the rainfall index method, which is analogous to the flood index method proposed by Dalrymple, (1960). We focus on the 25-yr, 50-yr, and 100-yr RIs for estimation of PI quantiles due to their significance and frequent application in the RSCS design discharge calculation, and ecological implications (Hecht et al. 2021; Jakob et al. 2020). Text S2 provides a detailed discussion on the methodology used for the GOF test and the estimation of precipitation intensity quantiles in this study.

The confidence intervals (CIs) associated with the quantile estimates were defined by 90% confidence level and calculated with Monte Carlo simulations (a 1,000 repeated samplings). A detailed discussion on the procedure followed for the estimation of the population parameters of the fitted distribution, regional and site-specific precipitation quantiles, and associated CIs is provided in Hosking and Wallis (1997). After the station-based PI quantiles were estimated, we applied the universal kriging interpolation methodology by using elevation of the Oregon region as covariate in the ‘‘*gstat*’’ R-package (Pebesma 2004) to spatially interpolate the PI quantiles across Oregon.

### 2.4 Onsite precipitation IDF analysis

Onsite precipitation IDF analysis was performed based on Extreme Value Analysis (EVA; Coles 2004) using the ‘‘*extRemes*’’ R-package (Gilleland and Katz 2016) for the AMS of PIs. In this study, the Onsite-PIs were evaluated for the 25-yr, 50-yr, and 100-yr RIs for the reason stated above. Onsite-PIs from the available record of the EF gauging stations (Table 1) were obtained for short (15-min and 30-min), intermediate (1-h, 2-h, and 3-h), and long (6-h, 12-h, and 24-h) duration storms. AMS of short-to-long duration storm intensities (or PIs) were derived using the Block Maxima (BM) methodology (AghaKouchak and Nasrollahi 2010; Hawkes et al. 2008; Katz et al. 2002; Li et al. 2015; Madsen et al. 1997).

In EVA theory, AMS is typically modeled by fitting a GEV distribution (Coles 2001; Nerantzaki and Papalexiou 2022; Emmanouil et al. 2020). The following section provides a detailed description of the AMS-based precipitation IDF analysis performed in the study.

In AMS based precipitation IDF analysis, the GEV distribution is fit to the AMS. The GEV is a three-parameter distribution comprised of the location (μ), scale(σ), and shape (ε) parameters whose theoretical cumulative distribution function is given as (Coles 2001),

(2) $$F_{GEV}(x|\mu ,\sigma ,\varepsilon )=exp\left[-\left(1+\frac{\varepsilon }{\sigma }{\left(x-\mu \right)}^{-1/\varepsilon }\right],\mu \right.\;\;\;\;\;\in R,\sigma >0,\varepsilon \neq 0$$

The *p*-quantile of the GEV distribution is then estimated as,

(3) $$q_{p}=\left[{\left(-\frac{1}{\ln(1-p)}\right)}^{\varepsilon }-1\right]\times \frac{\sigma }{\varepsilon }+\mu ,(\varepsilon \neq 0)$$

where, (1-p) is the non-exceedance probability.

In this study, the parameters of the GEV distributions were estimated based on L-moments methodology (Hosking et al. 1985). The L-moments methodology is shown to outperform the Method of Moments (Ossiander and Waymire 2000; Langousis and Veneziano 2007; Nerantzaki and Papalexiou 2022). In addition, the L-moments method is also consistent with the methodology adopted by NOAA-Atlas14 (Perica et al. 2018). The L-moments method has several advantages, especially in case of small sample size of precipitation series fitted to the EV distribution. The L-moments methodology also produces lower estimation bias and are less susceptible to outliers in extreme data as compared to other commonly used parameter estimation methodologies, such as maximum likelihood estimation, and method of moments (Nerantzaki and Papalexiou 2022).

The 10–90% confidence intervals (CIs) of the parameters are estimated with parametric bootstrapping from 1000 samples selected randomly using the ‘‘*extRemes*’’ R-package (Gilleland and Katz 2016).

### 2.5 Comparison between onsite and NOAA Precipitation IDFs

Precipitation IDF curves derived from the USDAFS onsite rain gauges were evaluated against NOAA precipitation IDFs. For HJA, the Onsite-IDFs were evaluated against the precipitation IDFs derived with RFA using NOAA’s hourly precipitation data (hereafter, referred as NOAA-RFA precipitation IDFs) because NOAA-Atlas14 PIs are unavailable for Oregon. In this study, the comparison of precipitation IDFs was performed by evaluating—Onsite-PI against NOAA PIs for a given duration and return interval (RI; frequency), and the onsite storm RI against NOAA’s 25-yr, 50-yr, and 100-yr RIs as a baseline. Detailed descriptions of these procedures are discussed below.

#### 2.5.1 Evaluation of onsite-PIs against NOAA

This evaluation was performed for all storm durations and frequencies selected in the study based on the percentage relative difference, which is defined as follows:

(4) $$RelativeDifference_{f,d}=\left(\frac{PI_{f,d}^{Onsite}-PI_{f,d}^{NOAA}}{PI_{f,d}^{NOAA}}\right)\times 100\text{\%}$$

where, f represents the frequency, e.g., 25-, 50-, and 100-yr RIs used here, and d denotes the storm durations for which the relative differences are calculated. This method is an adequate performance measure since it is normalized and, therefore, not sensitive to the absolute values of rainfall (Amatya et al. 2021; Ombadi et al. 2018)

#### 2.5.2 Evaluation of the onsite storm frequencies against NOAA

Precipitation storm frequencies (or RIs) for given durations, obtained from the onsite USDAFS rain gauge stations (Table 1) were evaluated against NOAA’s 25-, 50-, and 100-yr RIs (Perica et al. 2018). Onsite-PIs for specific durations were first calculated for RIs ranging between 1-yr and 500-yr. Finally, the Onsite-PI magnitudes that match with NOAA’s 25-, 50-, and 100-yr PI magnitudes were obtained and the corresponding RIs, derived from the onsite precipitation frequency estimates, were extracted. For example, a given precipitation storm event whose Onsite-PI magnitude amounts to the same as that of NOAA’s 100-yr PI is deemed to be more frequent based on the Onsite-IDF estimates, if the Onsite-PI corresponds to a RI < 100-yr. In that case, using NOAA’s 100-yr PIs will lead to undersizing of the RSCS, and potentially increase risks for structural and ecological failures (disruption of stream connectivity and aquatic passage). Importantly, previous studies have reported significant increases in the occurrence of heavy precipitation storm events in a changing climate (Cheng and AghaKouchak 2014; Fowler et al. 2021; Mamo 2015; Simonovic and Peck 2009). This type of comparison emphasizes the frequency of occurrence (the RI) of a storm event of a given intensity, providing an opportunity for forest land-managers and design engineers to choose between the Onsite, and NOAA precipitation IDFs specific to 25-yr, 50-yr, and 100-yr storm events, which are of main interest for design of RSCS in the NFLs. In addition, the evaluation also provides a test for the reliability of the stationarity assumption used in NOAA precipitation IDF estimation.

## 3 Results and discussion

### 3.1 Comparison between onsite-RFA and NOAA-RFA Precipitation IDFs for HJ andrews EF

A regional frequency analysis (RFA) was performed to estimate precipitation IDFs using NOAA’s hourly precipitation data from several stations located in Oregon due to unavailability of NOAA-Atlas14 precipitation IDF estimates for the State of Oregon (Perica et al. 2018). The location of NOAA’s rain gauge stations and identified homogenous regions used in the NOAA-RFA, and results from spatial interpolation of PI quantiles are illustrated in Fig. S7-S9 and Table S1 (Supplemental Information). The results of the heterogeneity test, and GOF test used for NOAA-RFA are presented in Table S2. Location, scale, and shape parameter of the fitted regional GEV distribution and site-specific scale parameters for the homogeneous regions (Region 1, 2, 3, and 4) selected from the available NOAA stations are shown in Table S3. AMS of PIs and linear trends in the PIs for all the selected stations are shown in Fig. S11-S20. The p-values obtained from Mann– Kendall trend test (α = 0.05) are also shown, where a trend is considered statistically significant if p-value (shown in red) is found to be less than 0.05. The AMS of PIs exhibit stationarity with non-significant trends for most or all storm durations at HJA-H15MET, HJA-UPLMET, CHL-RRG06, CHL-RRG41, CHL-RRG31, SAN-MET25, ALC-AC04, and FRS-HQTRS (Fig. S11-S20).

**Fig. 2:**
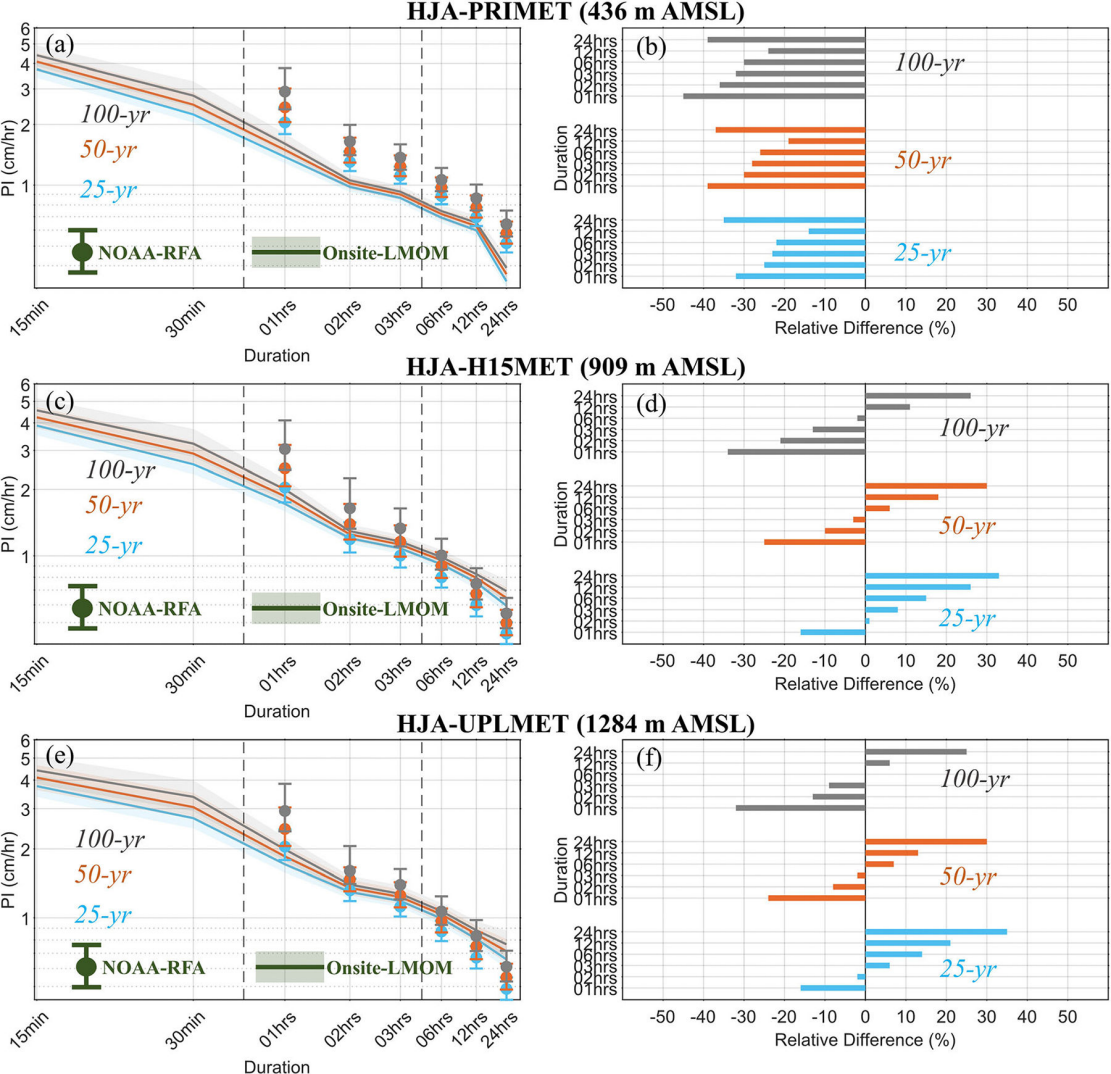
Precipitation IDF curves (left panel), and relative difference (%), shown by bar-plots, between Onsite-RFA and NOAA-RFA precipitation IDFs (right panel) for three selected rain gauge stations in HJA. The shading and error-bars in panels **a, c** and **e** indicate the 90% CIs of PIs estimated with the Onsite-RFA, and NOAA-RFA, respectively. The x- and y-axis of the precipitation IDF curves are on the logarithmic scales

Onsite precipitation IDFs were estimated using RFA considering the three selected stations within HJA, HJA-PRIMET, HJA-H15MET and HJA-UPLMET. The location, scale, and shape parameter of the fitted regional GEV distribution and site-specific scale parameters are shown in Table S4. The Onsite-RFA precipitation IDFs were compared to the NOAA-RFA precipitation IDFs at these three stations. Considerable disparities are evident between the Onsite-RFA and NOAA-RFA based precipitation IDF estimates (Fig. 2; Table 2, and Table S5-S7). Specifically, relative to the Onsite-RFA, the NOAA-RFA underestimate PIs of 25-yr, 50-yr, and 100-yr RIs for the longer duration (> 6-h) storms at the HJA-H15MET and HJA-UPLMET stations, and overpredicts for all durations at the lower elevation HJA-PRIMET station (Fig. 2). For example, at the HJA-H15MET station, NOAA-RFA underestimates PI of 25-yr, 50-yr, and 100-yr RIs by 26%, 18%, and 11% for the 12-h duration, and 33%, 30%, and 26% for the 24-h duration storm event. Similarly, at the HJA-UPLMET station, NOAA-RFA precipitation IDFs underestimate PI of 25-yr, 50-yr, and 100-yr RIs by 21%, 13%, and 6% for the 12-h duration, and 35%, 30%, and 25% for the 24-h duration storm event. Interestingly, the relative differences exhibit a similar dependence on storm durations for these two stations. At both stations, NOAA-RFA overpredicts PI for 25-yr, 50-yr, and 100-yr RIs for the shorter duration storms, and underpredicts for the longer duration storms with a shift in the inflection point from shorter (2-h) to longer (6-h) duration storms for increases in RIs from 25-yr to 100-yr (Fig. 2d, and f). On the other hand, at HJA-PRIMET stations, the NOAA-RFA overpredicts PIs for all duration storms and RIs. The uncertainty (shown by the 90% CIs) in Onsite-RFA precipitation IDFs are comparable with those of NOAA-RFA precipitation IDFs for the 25-yr, 50-yr, and 100-yr RIs (Fig. 2c). For shorter storm durations, the uncertainty range of PIs is narrower for the lower elevation station, HJA-PRIMET compared to the higher elevation stations, HJA-H15MET, and HJA-UPLMET which can be attributed to the longer data record available for the HJA-PRIMET station (1979–2018).

Overall, these results agree with the general types of storms that the Pacific Northwest region receives, much of which are large regional storm systems, with smaller percentage of precipitation coming from convective storms or short-term events (Greenland et al. 2003; Waichler and Wigmosta, 2003). About 70% of the available sixty-three NOAA stations in Oregon, used for the NOAA-RFA, are located at elevation lower than 500 m (Table S1). As such, it can be concluded that not only does NOAA-RFA underpredict the long duration PIs but also it does not capture well the regional storm PIs at higher elevation stations, HJA-H15MET (elevation of 909 m), and HJA-UPLMET (elevation of 1284 m). Therefore, to prevent from under-designing of culverts near these locations, use of Onsite-RFA based PIs should be prioritized in the higher elevation (> 500 m) stations, particularly for longer (> 6-h) duration storms for USDAFS RSCS design in the HJA EF.

### 3.2 Comparison between onsite-RFA and NOAA-Atlas14 precipitation IDFs for coweeta hydrological laboratory EF

**Fig. 3:**
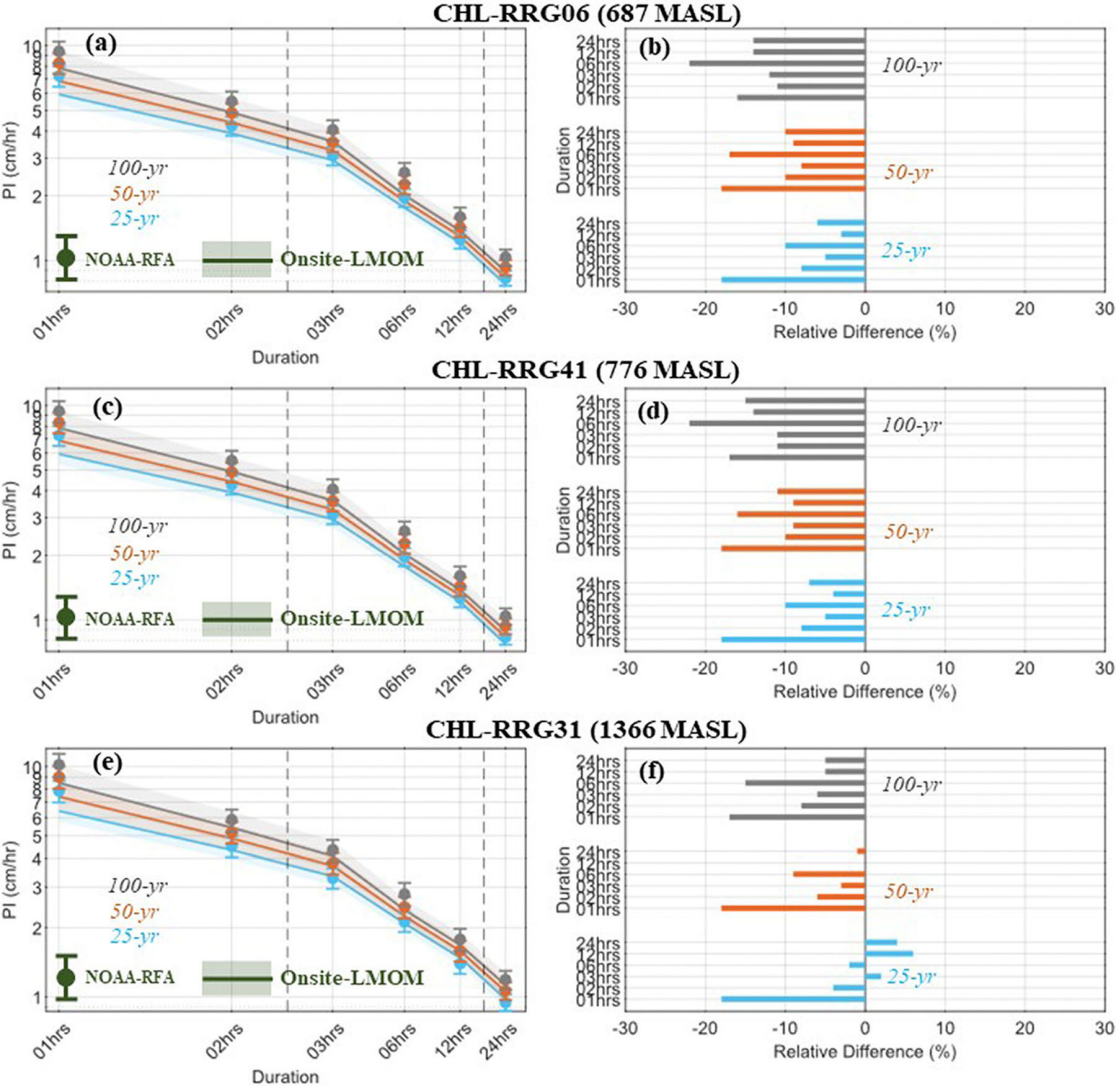
Precipitation IDF curves (left panel), and relative difference (%), shown by bar-plots, between Onsite-RFA and NOAA-Atlas14 precipitation IDFs (right panel) for three selected rain gauge stations in CHL. The shading and error-bars indicate the 90% CIs of PIs estimated with the Onsite-RFA, and NOAA-RFA, respectively. The x- and y-axis of the precipitation IDF curves are on the logarithmic scales

Onsite precipitation IDFs were estimated using RFA considering the three selected rain gauge stations in CHL, CHL-RRG06, CHL-RRG41, and CHL-RRG31. The location, scale, and shape parameter of the fitted regional GEV distribution and site-specific scale parameters are shown in Table S8. The Onsite-RFA precipitation IDFs were compared to the NOAA-Atlas14 precipitation IDFs at these three stations. There is little difference between the OnsiteRFA and NOAA-Atlas14 based precipitation IDF estimates for all three stations (Fig. 3; Table 2; and Table S9-S11). NOAA-Atlas14 slightly overpredicts the PIs at the CHL-RRG06, and CHL-RRG41 stations for all storm durations and RIs. On the other hand, NOAA-Atlas14 slightly underpredicts the PIs at the CHL-RRG31 station for longer duration storms (C 12-h) for the 25-yr and 50-yr RIs. For instance, at the CHL-RRG06 station, the PI of the 25-, 50-, and 100-year RIs is overpredicted by NOAA-Atlas14 by 18%, 18%, and 16% for storm events lasting 1-h, and by 6%, 10%, and 14% for storm events lasting 24-h. Similar to this, NOAA-Atlas14 precipitation IDFs at the CHL-RRG41 station overestimate PI of 25-, 50-, and 100-year RIs by 18%, 18%, and 17% for the 1-h duration, and by 7%, 11%, and 15% for the 24-h duration storm events (Fig. 3b, and 3d). It is interesting to note that both these stations are located within a 0.5-mile (CHL-RRG06) and 1-mile (CHL-RRG41) radius of the single available NOAA rain gauge station (COWEETA EXP STN) within the CHL EF region (Fig. S10). More importantly, these two stations are located at about the same elevation (CHL-RRG06: 687 m; and CHL-RRG41: 776 m) as the NOAA rain gauge station (685 m).

At the CHL-RRG31 station, however, NOAA-Atlas14 underpredicts PIs by 4–5% for 25-yr RI, and overpredicts by 1% and 5% for the 50-yr and 100-yr RIs for longer duration storms (C 12-h; Fig. 3f). These results indicate that the difference in Onsite-RFA and NOAA-Atlas14 PIs at the CHL-RRG31 station are very dissimilar from that observed at the CHL-RRG06, and CHL-RRG41 stations. This disparity may be due to the considerable difference in elevation of the CHL-RRG31 station (elevation of 1366 m) from the nearest (within 3-mile radius) available NOAA rain gauge station (COWEETA EXP STN: elevation of 776 m). The uncertainty (shown by the 90% CIs) in Onsite-RFA precipitation IDFs are comparable with those of NOAA-RFA precipitation IDFs for the 25-yr, 50-yr, and 100-yr RIs. The uncertainty range of PIs is, however, slightly narrower for the lower elevation stations, compared to the higher elevation station, CHL-RRG31.

It is important to note that these results are contrastingly different from the PIs estimated in Amatya et al., (2021) for the same rain gauge stations with the data used throughout the year 2015. This is perhaps due to the additional 7 years of data set (1976–2021) used in the current study for all three stations. The Onsite-PIs are found to increase for the higher elevation station during the most recent period (2016–2021; Fig. S14-S16). In addition, this study uses L-moment based parameter estimation methodology, whereas Amatya et al., (2021) used Bayesian parameter estimation to calculate the precipitation IDFs. L-moment based parameter estimates are found to be more robust for relatively smaller sample sizes (46-year period) compared to Bayesian parameter estimation methodology Nerantzaki and Papalexiou (2022). This is reflected in the narrower uncertainty range of PIs estimated in this study as compared to those obtained using the Bayesian parameter estimation methodology (Amatya et. al., 2021).

### 3.3 Comparison between onsite-LMOM and NOAA-Atlas14 precipitation IDFs for four EFs

**Fig. 4:**
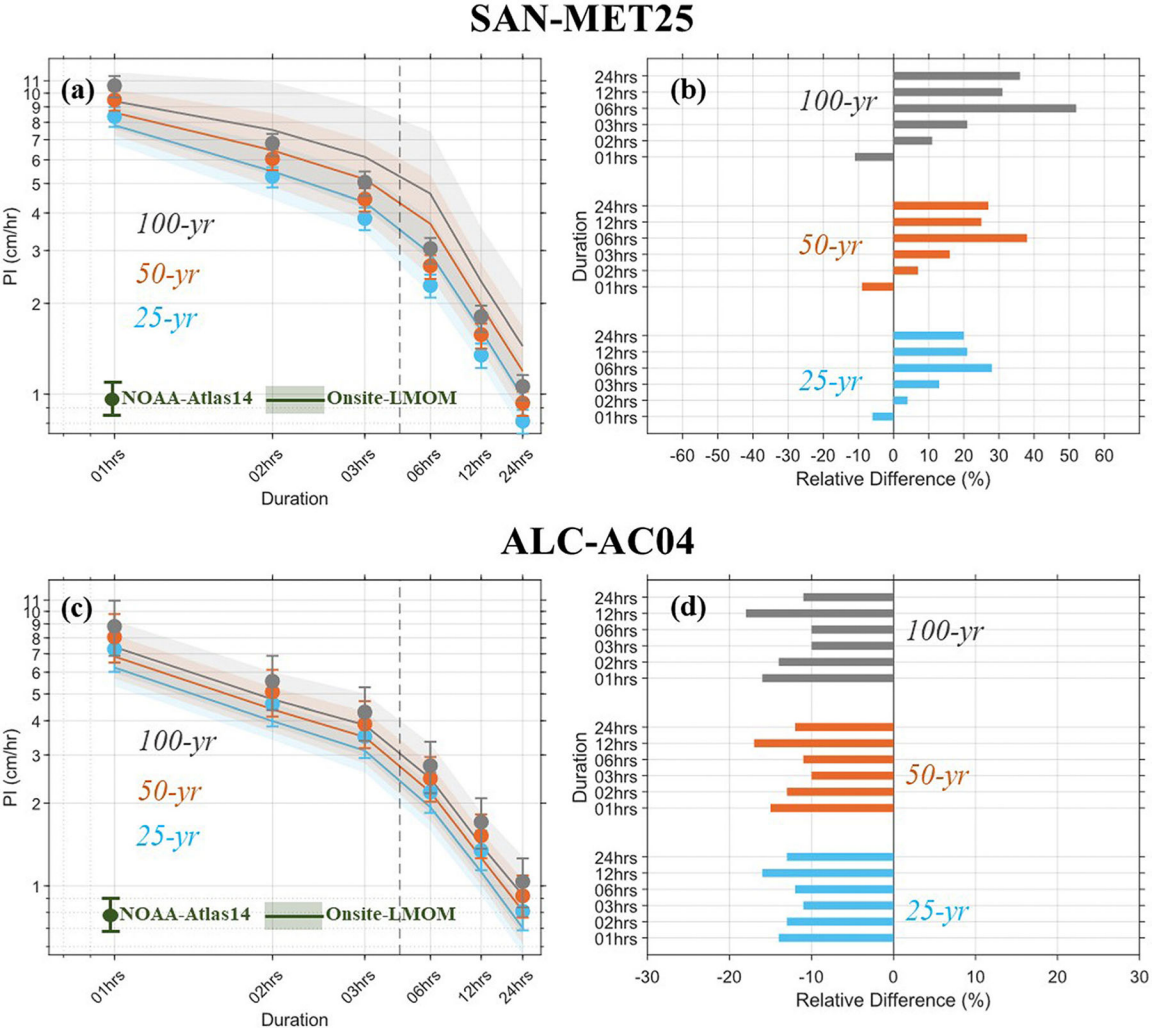
Precipitation IDF curves, and relative difference (in %; shown by bar plots) between Onsite-LMOM precipitation IDF and NOAA-Atlas14 precipitation IDF estimates at (**a-b**) SAN-MET25, and (c-d) ALC-AC04 station. The shading and error-bars in the precipitation IDF curves indicate the 90% confidence intervals of PIs corresponding to the Onsite-LMOM, and NOAA-Atlas14, respectively. The x-and y-axis of the precipitation IDF curves are on the logarithmic scales

**Fig. 5:**
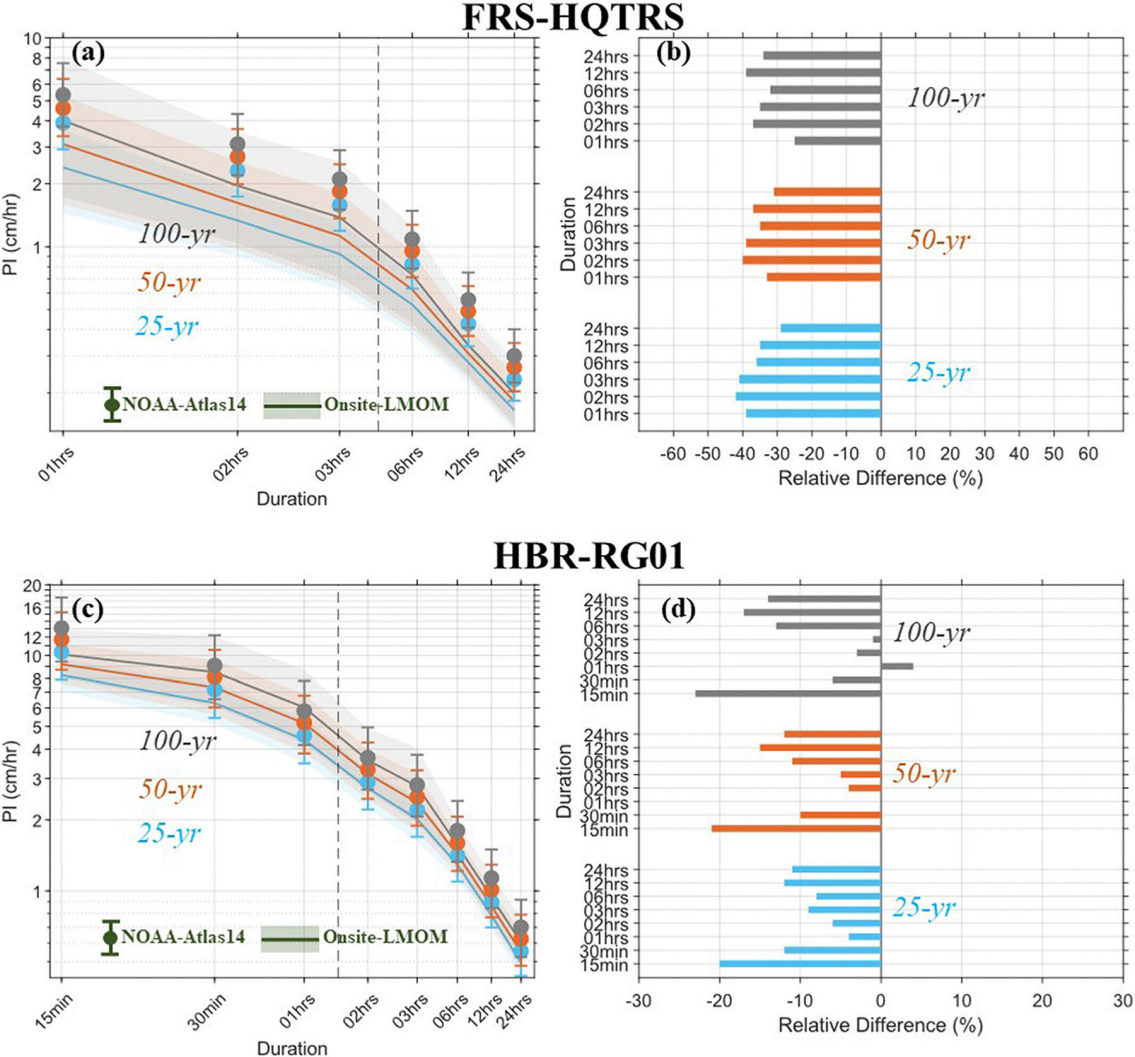
Precipitation IDF curves, and relative difference (in %; shown by bar plots) between Onsite-LMOM precipitation IDF and NOAA-Atlas14 precipitation IDF estimates at (**a-b**) FRS-HQTRS, and (**c-d**) HBR-RG01 station. The shading and error-bars in the precipitation IDF curves indicate the 90% confidence intervals of PIs corresponding to the Onsite-LMOM, and NOAA-Atlas14, respectively. The x-and y-axis of the precipitation IDF curves are on the logarithmic scales

precipitation IDFs estimated with Onsite-LMOM analysis for four USDAFS stations at Santee (SAN-MET25), Alum Creek (ALC-AC04), Fraser (FRS-HQTRS), and Hubbard Brook (HBR-RG01) EF are compared with precipitation IDFs obtained from NOAA-Atlas14, as shown in Figs. 4 and 5 (also refer to Table 2; Table S12-S19 for PIs and parameters of the GEV distribution). Relative differences between the PIs are also evaluated for these stations as shown by the bar-plots in Figs. 4 and 5.

Considerable disparities are evident between the Onsite-and NOAA-Atlas14 based precipitation IDFs estimates at multiple stations (Fig. 4). At the SAN-MET25 station, the uncertainties in PI estimates are considerably higher than in the NOAA-Atlas14 based PIs. NOAA-Atlas14 underestimates the PI for 25-yr, 50-yr and 100-yr RIs for all but 1-h duration storms. The relative differences between the PI for 1-h 25-yr, 50-yr, and 100-yr RIs are 5%, 9% and 11%, respectively, indicating an increasing trend for increasing RIs. At the ALC-AC04 station, the uncertainty range of the Onsite-PIs is comparable with those of NOAA-Atlas14, likely due to the record period of the NOAA-Atlas14 was through 2013, unlike only through 2006 for the SAN-MET25 station. However, NOAA-Atlas14 overpredicts PI for 25-yr to 100-yr RIs by only 5–11% for all duration storm events. For the FRS-HQTRS station, the uncertainty range of the Onsite-PIs is considerably higher than those of NOAA-Atlas14, which may be due to the limited number of years (18 years; 2004–2021 period) with data records available for this station (Fig. 5). Moreover, NOAA-Atlas14 overpredicts the PIs for 25-yr, 50-yr, and 100-yr RIs by more than 25% for all duration storm events. At the HBR-RG01 station, the uncertainty range of the Onsite-PIs is comparable with that of the NOAA-Atlas14 (Fig. 5). NOAA-Atlas14 PIs are also comparable with the Onsite-LMOM PIs for the 30-min, 1-h to 24-h durations and 25-yr, 50-yr, and 100-yr RIs. Except for the PI for 1-h 100-yr RI, NOAA-Atlas14 overpredicts the PIs for 25-yr to 100-yr RIs by 1% to 17% for the 30-min to 24-h storm durations.

The results of this study are in close agreement with a previous study that included similar analyses with the AMS data at the SAN-MET25 (ALC-AC04) stations and showed that NOAA-Atlas14 precipitation IDFs underestimated (overestimated) PIs when compared to estimates using onsite data only through 2016 from these stations and the percent differences increased for longer RIs and storm durations (Amatya et al. 2021). However, the relative differences in PIs from NOAA-Atlas14 and onsite data at SAN-MET25 were reported to be as high as 60% which is considerably higher compared to our results. Such disparities can be attributed to the difference in the length of data period and (L-moment based) parameter estimation methodology used in this study compared to that in Amatya (2021) which used relatively shorter duration of precipitation dataset and Bayesian parameter estimation methodology.

### 3.4 Comparison between onsite and NOAA precipitation frequency

**Fig. 6:**
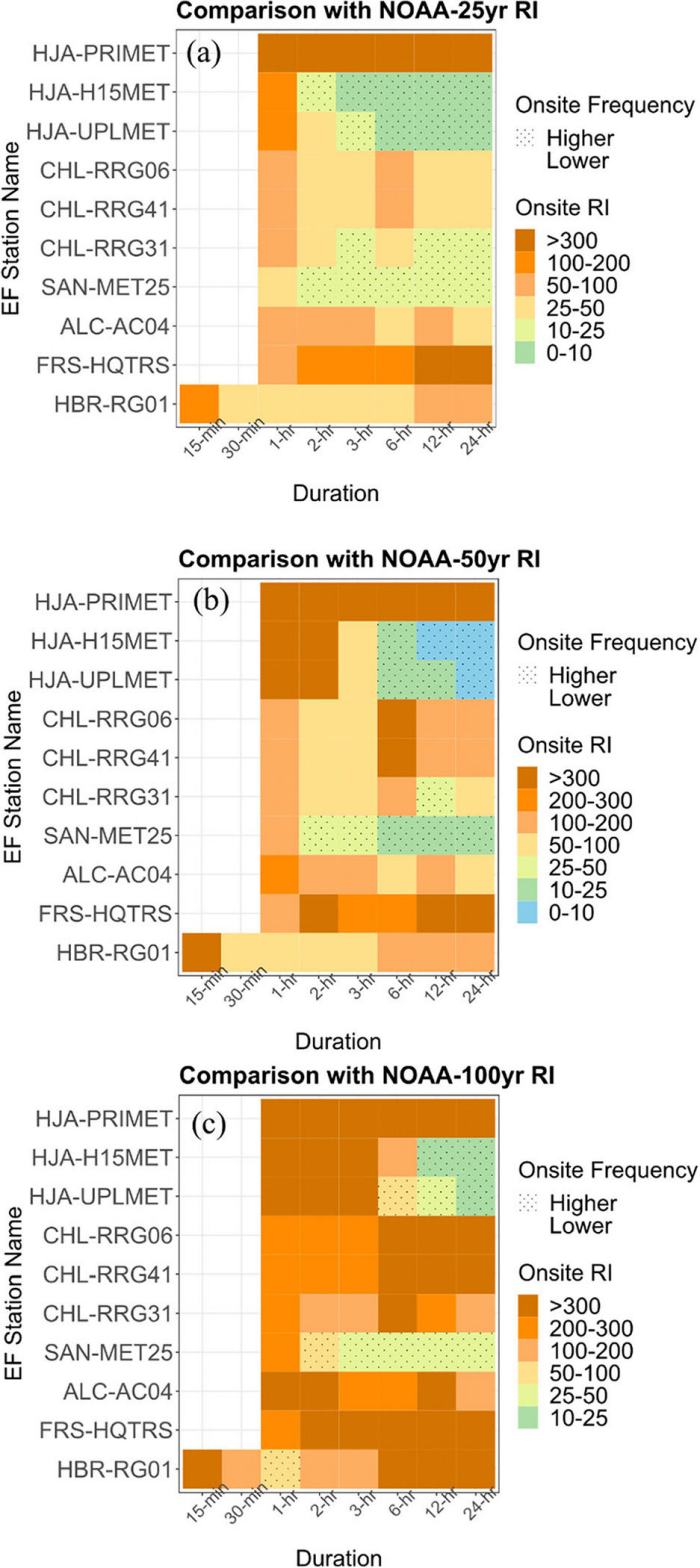
Comparison between NOAA-based RI (25-yr, 50-yr, and 100-yr) and Onsite-based storm frequency (RI; unit: year). Storm durations and stations for which the Onsite-LMOM and Onsite-RFA based RIs (Onsite frequency) are higher than those of NOAA-based RIs, are indicated by stippling

We evaluated the specific frequencies of PIs related to 25-yr, 50-yr, and 100-yr RIs provided by NOAA-RFA (for HJA) and NOAA-Atlas14 (for SAN, CHL, ALC, and HBR) against those estimated with the most updated (through year, 2021) USDAFS onsite rain gauge station dataset (based on Onsite-RFA for HJA, and CHL; and Onsite-LMOM for SAN, ALC, FRS, and HBR) used in this study (see Methods). The comparison for precipitation IDFs is shown in Fig. 6. For multiple stations, PIs of storm events with RIs of 25-yr, 50-yr, and 100-yr based on NOAA-RFA and NOAA-Atlas14 precipitation IDFs correspond to PIs of storm events with less than 25-yr, 50-yr, and 100-yr RI, respectively, based on Onsite-IDFs. For instance, at the HJA-H15MET and HJA-UPLMET stations, PIs of 3-h, 6-h, 12-h, and 24-h duration storms with 25-yr RI based on NOAA-RFA correspond to PIs of more frequent storm events with less than 25-yr (10-yr) RI based on Onsite-RFA estimated precipitation IDFs (Fig. 6a). At the higher elevation station, CHL-RRG3, PIs of 3-h, 12-h and 24-h with 25-yr RI based on NOAA-Atlas14 correspond to PIs of storm events with less than 25-yr RI based on Onsite-RFA estimated precipitation IDFs. At the SAN-MET25 station, PIs of 2-h to 24-h specific to 25-yr RI based on NOAA-Atlas14 correspond to PIs of storm events with less than 25-yr RI based on Onsite-LMOM estimated precipitation IDFs, consistent with Amatya et al., (2021). Similarly, PIs of 12-h and 24-h storm events with 100-yr RIs correspond to PIs of more frequent storm events with 10–25-yr RI at HJA-H15MET; 6-h, 12-h and 24-h events with 100-yr RI correspond to events with 50- to 100-yr, 25-to 50-yr and 10- to 25-yr RI, respectively, at HJAUPLMET; 2-h, and 3- to 24-h events with 100-yr RIs correspond to events with less than 100-yr, and 25–50-yr RI at SAN-MET25; and 1-h events with 100-yr RI correspond to events with less than 100-yr RI at the HBR-RG01 station.

## 4 Summary and conclusions

We evaluated Onsite precipitation Intensity-Duration-Frequencies (referred to as Onsite-IDFs) using 40 ? years of high resolution precipitation data at six long-term USDA Forest Service (USDAFS) Experimental Forests (EFs) against the precipitation IDFs published by NOAA (NOAA-Atlas14 precipitation IDFs; Perica et al., (2018)). Based on the results of this analysis we suggested/recommended the use of the NOAA PIs or more local onsite and regional precipitation IDF measurements, where possible, in hydrologic models for determining associated design storm discharge rates from watersheds. This research is motivated by the recent increase in extreme precipitation events, which have the potential to threaten forest road cross drainage structures and, for that matter disrupt transportation as well as ecological systems. We hypothesized added benefits and guidelines of using Onsite-IDF over the NOAA-Atlas14 precipitation IDFs, where applicable in this study, which can aid in the design, and planning of resilient road crossing infrastructure at head-water forest catchments. Accordingly, this study attempted to evaluate the precipitation IDFs based on annual maxima series (AMS) for onsite sub-daily and daily precipitation intensities (PIs) by comparing them with precipitation IDF estimates provided by NOAA.

Considerable disparities were observed between the onsite and published NOAA Atlas-14 precipitation IDFs. In the case of the precipitation IDFs estimated with Onsite-RFA, these disparities in PIs exhibited their strong dependence on the storm durations and elevation of stations, particularly in Horace Justin Andrews (HJA) and Coweeta Hydrological Laboratory (CHL) EFs. Onsite PIs estimated for higher elevation stations were greater than PIs of low-elevation stations for all duration storm events, and were greater than NOAA-Atlas14’s PI estimates for longer duration storm events. This contrasts with what Amatya et al. (2021) found earlier in the CHL EF. Amatya et al. (2021) used data up to 2015 to estimate precipitation IDFs while the last seven years of the period 1976–2021, used in this study, saw an increase in PIs at the higher elevation station compared to the lower elevation stations of CHL. Additionally, this study used the L-moment, a different approach of parameter estimation as compared to Amatya et al. (2021), which is more reliable for fitting data with small sample sizes and is widely used in NOAA-Atlas14. This is reflected in the Onsite-IDFs computed in this work, which have a lower uncertainty range.

On the other hand, some of our findings are consistent with those of Amatya et al. (2021), who utilized onsite data from the low-gradient SAN EF up until 2015 and reported a similar underprediction of NOAA-Atlas14 PIs, particularly for longer duration storm events. The return interval of storms with identical PIs was also examined in this study between precipitation IDFs estimated using onsite data and those provided by the NOAA-Atlas14. It has been noted that storms with the same PIs and longer durations reported by NOAA-Atlas14 are estimated to show higher frequency or lower RI estimates based on the Onsite-LMOM and Onsite-RFA at HJA-H15MET, HJA-UPLMET, and SAN-MET25 stations. At the majority of other USDAFS rain gauges and storm durations, NOAA overpredicts the PIs. Therefore, design discharge estimation at those sites should be done using NOAA-based PIs, even if this can result in a minor overdesign of the RSCS. However, somewhat overdesigning the RSCS for flood protection is reasonable to ensure that the structure is able to withstand the potentially unforeseen increase in future flood event, which can lead to property damage and other ecologic and economic losses that can be often much higher than any overdesign costs. Additionally, overdesigning an RSCS to some possible extent can help to maximize its lifespan, allowing it to provide protection for many years to come.

The increase in anthropogenically caused climate change, which has the potential to disrupt the stationarity of the precipitation series, necessitates annual adjustments to the precipitation IDF estimations (Easterling et al. 2017). This is an important caveat that should be taken into consideration with the upcoming NOAA-Atlas14 historical precipitation IDF updates as well as updates that would consider future rainfall data derived from downscaled global climate models (e.g., Jalowska et al., (2021)). However, it should be noted that Extreme Value Analysis demands more research on the selection of covariates in the estimation of distribution parameters, especially in complex mountainous terrains such as H. J. Andrews, Fraser, and Hubbard Brook EFs, and eastern temperate forests, such as in Santee and Coweeta EFs, where extreme precipitation storms led by tropical storms/hurricanes and atmospheric rivers are a common phenomenon (Dhakal and Jain 2020; Mukherjee and Mishra 2021; Ren et al. 2019). One of the scopes of further improvements in this study will be to reduce the statistical variability that leads to the coexistence of negative and positive shape parameter estimates (upper bound) which defies the physical nature of rainfall extremes (Emmanouil et al. 2020). Furthermore, to deal with challenges arising from limited spatial coverage of gauges and the complex heterogeneity of precipitation, especially in the mountainous terrain (Basist et al. 1994; Preece et al. 2021; Wu et al. 2020; Yu et al. 2020) OnsiteIDFs can be evaluated against precipitation IDFs derived using satellite-based rainfall products (Ciabatta et al. 2016) and precipitation estimates deduced from soil moisture measurements (Brocca et al. 2013), which will certainly be another future scope of this work. Most of the USDAFS rain gauge’s long-term high-resolution datasets, such as those used in this manuscript as well as the remaining stations, are still undergoing digitization from paper charts recorded for historic precipitation dataset, which is resource intensive (Amatya et al. 2021). We used our best efforts to use data by digitizing the historic charts-based data for three of the six stations used in this study. As a future scope of the work, we intend to use a greater number of rain gauges across many USDAFS lands.

Overall, the results of this study highlight the value of long-term high-resolution precipitation records from the USDAFS Experimental Forest network of rain gauges for evaluating extreme PIs. Many EF sites, beyond those examined here, have long-term data that would be more appropriate for analyses, needing onsite climate data even though historical charts have to be digitized in some cases as was done for this study. The use of those data will be more reliable than the use of data from more distant NOAA stations or from different topography. Given the study’s findings, the authors recommend using Onsite-IDF estimates, especially for longer duration storm events, in rainfall-runoff and hydrologic models, used for estimating design storm discharge rates to size resilient road culverts and crossings at higher elevation catchments in HJA, CHL EF, and low-gradient ones in SAN EF.

## Supplementary Material

SupplementaryMaterial

**Supplementary Information** The online version contains supplementary material available at https://doi.org/10.1007/s00477–023-02495–0.

## Figures and Tables

**Table 1:** Onsite rain gauge coordinate locations, elevations (AMSL—above mean sea level), available rainfall data period, and their temporal resolution for the six EFs included in the study

Name of EF (or Study Sites)	USDAFS Station ID (abbreviation used in the manuscript)	Time-period used for the study			Finest temporal resolution available	Coordinates (degrees)		Elevation (AMSL)
		Start year	End year	Number of years		Longitude (W)	Latitude (N)
HJ Andrews (HJA)	PRIMET (HJA-PRIMET)	1979	2018	40	5-min	– 122.255941	44.211893	436
	H15MET (HJA-H15MET)	1984	2018	35	5-min	– 122.173782	44.26425	909
	UPLMET01 (HJA-UPLMET)	1995	2018	24	5-min	– 122.119763	44.207097	1284
Coweeta	RRG06 NC (CHL-RRG06)	1976	2021	46	1-h	– 83.4301	35.060411	687
Hydrologic Lab. (CHL)	RRG41 NC (CHL-RRG41)	1976	2021	46	1-h	– 83.4287	35.055308	776
	RRG31 NC (CHL-RRG31)	1976	2021	46	1-h	– 83.468122	35.032747	1366
Santee (SAN)	MET25 (SAN-MET25)	1977	2021	45	1-h	– 79.883	33.1488	6
Alum Creek (ALC)	AC04 (ALC-AC04)	1976	2015	40	1-h	– 93.0245	34.70672	300
Fraser (FRS)	HQTRS (FRS-HQTRS)	2004	2021	18	1-h	– 105.883897	39.904814	2743
Hubbard Brook (HBR)	RG01 (HBR-RG01)	1977	2021	45	15-min	– 71.724837	43.95212	478

**Table 2:** Onsite, and NOAA-based 1-h and 24-h PIs for 25-yr, 50-yr, 100-yr return intervals

Name of EF	USDAFS station ID	1-h PI (cm/hr)			24-h PI (cm/hr)		
		25-yr	50-yr	100-yr	25-yr	50-yr	100-yr
HJA	HJA-PRIMET	1.38 (1.28, 1.5)	1.5 (1.37, 1.65)	1.6 (1.44, 1.8)	0.33 (0.31, 0.37)	0.36 (0.33, 0.4)	0.38 (0.34, 0.44)
		2.04 (1.79, 2.43)*	2.43 (2.05, 3.01)*	2.9 (2.37, 3.79)*	0.5 (0.46, 0.57)*	0.57 (0.51, 0.66)*	0.64 (0.55, 0.75)*
	HJA-H15MET	1.72 (1.59, 1.87)	1.86 (1.7, 2.04)	2 (1.78, 2.23)	0.59 (0.54, 0.66)	0.65 (0.58, 0.72)	0.69 (0.62, 0.78)
		2.04 (1.75, 2.47)*	2.5 (2.06, 3.17)*	3 (2.44, 4.1)*	0.45 (0.4, 0.5)*	0.5 (0.44, 0.57)*	0.55 (0.47, 0.64)*
	HJA-UPLMET	1.71 (1.58, 1.88)	1.85 (1.68, 2.07)	1.99 (1.77, 2.25)	0.66 (0.6, 0.74)	0.71 (0.64, 0.81)	0.77 (0.67, 0.88)
		2.05 (1.79, 2.43)*	2.45 (2.06, 3.04)*	2.93 (2.39, 3.86)*	0.49 (0.44, 0.55)*	0.55 (0.49, 0.63)*	0.61 (0.53, 0.72)*
CHL	CHL-RRG06	5.94 (5.33, 6.69)	6.85 (5.99, 7.95)	7.86 (6.67, 9.45)	0.77 (0.72, 0.84)	0.83 (0.76, 0.92)	0.89 (0.79, 1)
		7.24 (6.45, 8)*	8.31 (7.39, 9.22)*	9.4 (8.28, 10.44)*	0.83 (0.76, 0.89)*	0.93 (0.85, 1.01)*	1.04 (0.94, 1.13)*
	CHL-RRG41	5.93 (5.3, 6.67)	6.85 (5.96, 7.93)	7.86 (6.64, 9.37)	0.77 (0.71, 0.84)	0.83 (0.75, 0.92)	0.89 (0.78, 1.01)
		7.26 (6.48, 8.03)*	8.36 (7.42, 9.27)*	9.42 (8.31, 10.49)*	0.83 (0.76, 0.9)*	0.93 (0.86, 1.01)*	1.04 (0.95, 1.13)*
	CHL-RRG31	6.41 (5.769, 7.23)	7.4 (6.51, 8.54)	8.49 (7.26, 10.11)	0.98 (0.9, 1.07)	1.05 (0.95, 1.17)	1.12 (1, 1.27)
		7.82 (6.99, 8.66)*	9.02 (8, 10.03)*	10.19 (8.97, 11.35)*	0.94 (0.87, 1.02)*	1.06 (0.97, 1.16)*	1.19 (1.07, 1.3)*
SAN	SAN-MET25	7.81 (6.77, 8.97)	8.61 (7.23, 10.28)	9.4 (7.6, 11.73)	0.98 (0.75, 1.26)	1.19 (0.84, 1.67)	1.44 (0.93, 2.22)
		8.36 (7.72, 8.99)*	9.5 (8.74, 10.21)*	10.59 (9.68, 11.4)*	0.81 (0.74, 0.89)*	0.93 (0.85, 1.02)*	1.06 (0.96, 1.16)*
ALC	ALC-AC04	6.24 (5.33, 7.21)	6.84 (5.69, 8.19)	7.41 (5.97, 9.25)	0.7 (056, 0.86)	0.81 (0.61, 1.05)	0.93 (0.66, 1.28)
		7.29 (6.02, 8.71)*	8.05 (6.50, 9.78)*	8.81 (6.88, 10.95)*	0.81 (0.69, 0.95)*	0.92 (0.77, 1.09)*	1.04 (0.84, 1.26)*
FRS	FRS-HQTRS	2.39 (1.44, 3.61)	3.09 (1.59, 5.26)	4.02 (1.73, 7.88)	0.16 (0.13, 0.21)	0.18 (0.14, 0.24)	0.2 (0.14, 0.29)
		3.91 (2.92, 5.31)*	4.60 (3.38, 6.35)*	5.33 (3.76, 7.54)*	0.23 (0.18, 0.30)*	0.26 (0.2, 0.35)*	0.3 (0.22, 0.40)*
HBR	HBR-RRG01	4.39 (3.52, 5.43)	5.16 (3.89, 6.86)	6.02 (4.22, 8.71)	0.49 (0.43, 0.57)	0.55 (0.45, 0.66)	0.6 (0.48, 0.77)
		4.57 (3.48, 5.87)*	5.18 (3.84, 6.76)*	5.82 (4.17, 7.8)*	0.55 (0.43, 0.69)*	0.62 (0.48, 0.79)*	0.7 (0.52, 0.92)*

The numbers in the parenthesis indicate the 90% confidence intervals of PIs and the NOAA-based PIs are denoted with asterisks
