# Supplementary material for: Comparison of on-site versus NOAA’s extreme precipitation intensity-duration-frequency estimates for six forest headwater catchments across the continental United States
Source: Stoch Environ Res Risk Assess. Author manuscript; Available in PMC 2026 May 5. (PMC13137446; doi:10.1007/s00477-023-02495-0)
Supplement: SupplementaryMaterial [file NIHMS2156300-supplement-SupplementaryMaterial.docx]

**Supplementary Information**

**Comparison of On-site versus NOAA’s Extreme Precipitation Intensity-Duration-Frequency Estimates for Six Forest Headwater Catchments across the Continental United States**

**Sourav Mukherjee^1*^, Devendra M. Amatya^1^, Anna M. Jalowska^2^, John L. Campbell^3^, Sherri L. Johnson^4^, Kelly Elder^5^, Sudhanshu Panda^6^, Johnny M. Grace^7^, Duncan Kikoyo^8^**

^1^Center for Forested Wetlands Research, Southern Research Station, USDA Forest Service, 3734 Highway 402, Cordesville, SC 29434, USA.

^2^Office of Research and Development, U.S. Environmental Protection Agency, Research Triangle Park, NC, USA.

^3^Northern Research Station, USDA Forest Service, Durham, New Hampshire, USA.

^4^Pacific Northwest Research Station, USDA Forest Service, Corvallis, OR, USA.

^5^Rocky Mountain Research Station, USDA Forest Service, Fort Collins, Colorado, USA;

^6^Institute of Environmental Spatial Analysis, University of North Georgia, 3820 Mundy Mill Road, Oakwood, GA 30566, USA.

^7^Center for Forest Watershed Research, Southern Research Station, USDA Forest Service, 1740 S. Martin Luther King Jr. Blvd., Perry-Paige Bldg., Suite 303 North, Tallahassee, FL 32307, USA.

^8^Texas A&M University, College Station, USA.

^*^Corresponding author: Sourav Mukherjee ([sourav.mukherjee@usda.gov)](mailto:sourav.mukherjee@usda.gov))

**Contents**

**Text S1-S2**

**Tables S1 to S19**

**Figures S1 to S14**

**Test S1. Selection of homogeneous regions**

The selection of homogeneous region involved a cluster analysis followed by a heterogeneity test as explained below:

*Cluster Analysis:* We used Ward’s hierarchical clustering algorithm (Murtagh and Legendre, 2014) to identify preliminary homogeneous regions. Ward’s method employs a minimum variance criterion based on the sum of squares index to determine the distance between sites and clusters. A combination of different site characteristics was used for each rain gauge station as attributes for the cluster analysis, including location (latitude and longitude), elevation, long-term mean daily precipitation, and long-term mean monthly precipitation. Similar attributes were used in a previous study for identifying homogeneous regions in California and Florida (Srivastava et al., 2019).

*Test for heterogeneity:* Three measures of heterogeneity, *H_n_* (n=1,2,3) were used to verify the homogeneity of the clusters based on the observed and simulated dispersion of L-moments for the group of sites under consideration. *H_1_* compares the dispersion of sample L-CV estimates for a cluster with the dispersion of L-CV estimates for a realization (simulation) of a homogeneous region. The dispersion of sample L-CV (Vs) is defined as the weighted (by the record length at each station in a cluster) standard deviation of sample L-CV estimates. The L-CV estimates for a realization were calculated with Monte Carlo simulation. In each simulation, a homogeneous region was constructed using the same number of sites and record length as in the observed sample. The mean (µ_r_) and standard deviation (σ_r_) of the realizations were calculated from repeated Monte Carlo simulations (Hosking and Wallis 1997; Srivastava et al., 2019). Finally, the heterogeneity measure *H_1_* was calculated as

$$H_{1}=\frac{Vs-\mu_{r}}{\sigma_{r}} (1)$$

Thus, a larger positive value indicates that the observed L-moments are more dispersed than what is consistent with the hypothesis of homogeneity.

*H_2_* indicates whether the onsite and regional estimates are close to each other. A large value of *H_2_* indicates a large deviation between regional and onsite estimates. Finally, *H_3_* indicates whether the onsite and the regional estimates agree. Large values of *H_3_* suggest a large deviation between onsite estimates and observed data. A region is considered “acceptably homogeneous” if *H_n_* < 1; “possibly heterogeneous” if 1< *H_n_* < 2; and “definitely heterogeneous” if *H_n_* > 2. This criterion has been used in previous studies (Bonnin et al., 2006; Hosking and Wallis, 1997; Ngongondo et al., 2011; Srivastava et al., 2019); however, *H_1_* is the primary measure for the heterogeneity test as both H_2_ and H_3_ rarely yield values larger than 2 even for grossly heterogeneous regions (Hosking and Wallis, 1997; Yang et al., 2010). We accepted a cluster as homogeneous when *H_1_* yielded a value less than 2, even if there were a few discordant sites (Srivastava et al., 2019). A detailed explanation of the computational methods used for the calculation of *H_n_* is provided in Hosking and Wallis (1997).

**Text 2 Goodness-of-fit (GOF) test and estimation of precipitation intensity quantiles**

***Goodness-of-fit (GOF) test***

The GEV distribution was first fitted to the AMS from all NOAA sites within a study area. Finally, the GOF for the GEV distribution was tested using the Z-statistic described by Hosking & Wallis (1997). The Z-statistic measures how well the L-kurtosis of the fitted distribution matches the regional average L-kurtosis of the sample data, and given as (Hosking and Wallis 1997),

$$Z=\frac{\tau_{4}-t_{4}+B_{4}}{\sigma_{4}} (2)$$

where, $\tau_{4}$is the theoretical L-kurtosis of the fitted distribution, $t_{4}$is the regional average L-kurtosis estimated by weighting the site-specific L-kurtosis by the sample size, σ_4_ is the estimate of the standard deviation of $t_{4}$, and is evaluated by repeated Monte Carlo simulations, *B*_4_ is the bias correction term added to correct the bias of sample L-kurtosis *t*_4_, and is computed from the same Monte Carlo simulation used to calculate *σ*_4_. The GEV distribution was considered sufficient for fitting the AMS if it satisfied |Z | ≤ 1.64 (Hosking and Wallis 1997).

***Estimation of precipitation intensity quantiles***

First, the parameters of the fitted distribution, ($\hat{\theta}_{k}, k=1,2,..K)$, were estimated by equating the population L-moments of the fitted distribution to the regional average L-moment ratios. The regional quantiles, q(F), were then estimated by the inverse function of F (non-exceedance probability) as, $\hat{q}\left( F \right)=q(F; \hat{\theta}_{1},\hat{\theta}_{2},..\hat{\theta}_{k})$, also known as the growth curve. Finally, the AMS-based site-specific precipitation quantile at a site, *i* (*i = 1,2, …N*), and for a given RI (in year), was computed using the rainfall index method shown by equation (3) below.

$$Q_{i}\left( F \right)=\mu_{i}q\left( F \right) (1)$$

where, $\mu_{i}$ is the index-rainfall calculated as the mean of the on-site rainfall series, at site, *i*, and $q\left( F \right)$ is a dimensionless quantile function also known as the regional growth curve estimated with the RFA.

**Tables**

**Table S1** List of 63 NOAA 1-hr precipitation stations in Oregon selected for the NOAA-RFA for this study, including station ID, elevation, and location coordinates.

| **NOAA Station ID** | **Elevation (m)** | **Longitude** | **Latitude** | **NOAA Station ID** | **Elevation (m)** | **Longitude (ºW)** | **Latitude**  **(ºN)** |
| --- | --- | --- | --- | --- | --- | --- | --- |
| COOP101018 | 865.6 | -116.05 | 43.525 | COOP353915 | 380.1 | -122.43 | 43.712 |
| COOP101022 | 860.5 | -116.24 | 43.567 | COOP354008 | 116.7 | -121.55 | 45.655 |
| COOP103143 | 469.4 | -115.54 | 46.1 | COOP354238 | 67.1 | -123.01 | 44.723 |
| COOP105241 | 435.8 | -117.02 | 46.375 | COOP354276 | 173.7 | -123.53 | 45.941 |
| COOP105708 | 1531.6 | -116.1 | 44.887 | COOP354321 | 1338.1 | -117.05 | 42.979 |
| COOP350694 | 1115.6 | -121.28 | 44.057 | COOP354622 | 839.7 | -118.07 | 45.317 |
| COOP350781 | 295.7 | -123.07 | 43.595 | COOP354824 | 199.6 | -123.52 | 45.593 |
| COOP350897 | 18.9 | -121.95 | 45.635 | COOP355050 | 217 | -122.76 | 43.914 |
| COOP351033 | 298.1 | -122.05 | 45.385 | COOP355055 | 481.6 | -122.67 | 42.672 |
| COOP351222 | 106.7 | -123.19 | 45.69 | COOP355206 | 12.5 | -123.88 | 44.062 |
| COOP351643 | 6.7 | -123.21 | 46.108 | COOP355213 | 166.1 | -122.87 | 44.171 |
| COOP351735 | 204.2 | -122.43 | 45.173 | COOP355221 | 754.4 | -121.95 | 44.612 |
| COOP351765 | 878.1 | -120.18 | 45.241 | COOP355969 | 6.7 | -123.89 | 45.715 |
| COOP351902 | 253.3 | -123.06 | 43.718 | COOP356546 | 452.5 | -118.83 | 45.697 |
| COOP351914 | 384 | -122.24 | 44.131 | COOP357127 | 157 | -122.91 | 45.303 |
| COOP352292 | 371.9 | -122.25 | 44.724 | COOP357554 | 1143 | -121.94 | 44.435 |
| COOP352295 | 624.5 | -123.05 | 42.812 | COOP357572 | 12.2 | -122.83 | 45.656 |
| COOP352345 | 371.2 | -122.74 | 43.708 | COOP357631 | 705.6 | -122.53 | 44.953 |
| COOP352348 | 435.9 | -122.93 | 45.699 | COOP357823 | 124.4 | -122.77 | 45.006 |
| COOP352374 | 249.9 | -122.96 | 43.782 | COOP358182 | 227.4 | -123.58 | 44.637 |
| COOP352408 | 225.9 | -123.42 | 43.784 | COOP358263 | 410.6 | -123.06 | 43.422 |
| COOP352697 | 670.6 | -121.97 | 45.077 | COOP358466 | 341.4 | -122.07 | 45.122 |
| COOP353047 | 167.6 | -122.67 | 44.414 | COOP358504 | 128 | -123.6 | 45.413 |
| COOP353232 | 1484.4 | -121.13 | 42.205 | COOP358512 | 762.6 | -122.7 | 43.007 |
| COOP353305 | 424 | -123.43 | 42.743 | COOP358790 | 558.4 | -122.6 | 43.482 |
| COOP353318 | 196.3 | -123.31 | 45.656 | COOP358884 | 190.5 | -123.19 | 45.865 |
| COOP353340 | 161.5 | -122.93 | 45.99 | COOP359213 | 585.8 | -118.43 | 45.821 |
| COOP353402 | 1213.1 | -121.74 | 45.301 | COOP451160 | 345.6 | -121.97 | 45.868 |
| COOP353421 | 120.4 | -123.62 | 45.052 | COOP451759 | 158.5 | -122.35 | 46.009 |
| COOP353521 | 94.2 | -122.42 | 45.483 | COOP452030 | 474.6 | -118 | 46.315 |
| COOP353604 | 808.6 | -117.11 | 44.877 | COOP453183 | 566.9 | -121.26 | 46.008 |
| COOP353705 | 230.4 | -123.35 | 45.313 |  |  |  |  |

**Table S2** Heterogeneity Test Statistics (*H_n_*) and Goodness-of-fit Test Statistics (*Z*) for selected Homogenous Regions used in NOAA-RFA for Oregon stations in this study. H_n_ >1.0 suggests that further subdivision of the region should be considered. Given the region homogeneity, the fit may be acceptable at Z<1.645 in absolute value (Hosking and Wallis, 1997).

| **Homogenous Region** | **Heterogeneity Test Statistics (*H_n_*)** | | | **Goodness-of-fit Test Statistics (*Z*)** | | | | |
| --- | --- | --- | --- | --- | --- | --- | --- | --- |
|  | ***H_1_*** | ***H_2_*** | ***H_3_*** | ***Z.glo*** | ***Z.gev*** | ***Z.gno*** | ***Z.pe3*** | ***Z.gpa*** |
| 1 | -0.47 | -0.47 | -0.33 | 0.89 | -0.22 | -1.04 | -2.48 | -3.22 |
| 2 | -0.53 | -0.51 | -0.45 | 1.57 | -0.11 | -1.20 | -3.15 | -4.54 |
| 3 | -0.33 | -0.29 | -0.19 | 1.03 | 0.00 | -0.65 | -1.82 | -2.70 |
| 4 | -0.69 | -0.69 | -0.59 | 2.29 | -0.11 | -1.53 | -4.09 | -6.33 |

**Table** **S3** Location, scale, and shape parameters of the fitted regional GEV distribution and site-specific scale parameters for the homogeneous regions (Region 1, 2, 3, and 4) selected from the Oregon NOAA station chosen for the NOAA-RFA analysis for this study.

| **Region ID** | | **Region 1** | | | | | |
| --- | --- | --- | --- | --- | --- | --- | --- |
| **Duration** | | **1-hr** | **2-hr** | **3-hr** | **6-hr** | **12-hr** | **24-hr** |
| **Parameters of Fitted Regional GEV Distribution** | Location | 0.78 | 0.82 | 0.84 | 0.85 | 0.85 | 0.85 |
|  | Scale | 0.24 | 0.24 | 0.23 | 0.25 | 0.25 | 0.27 |
|  | Shape | -0.25 | -0.14 | -0.12 | -0.02 | -0.03 | 0.01 |
| **Site-specific Scale Parameter** | COOP101018 | 0.87 | 0.52 | 0.38 | 0.24 | 0.15 | 0.09 |
|  | COOP101022 | 0.73 | 0.45 | 0.34 | 0.21 | 0.14 | 0.08 |
|  | COOP351765 | 0.91 | 0.58 | 0.43 | 0.26 | 0.16 | 0.09 |
|  | COOP352697 | 0.98 | 0.64 | 0.52 | 0.38 | 0.28 | 0.20 |
|  | COOP353604 | 1.02 | 0.60 | 0.46 | 0.30 | 0.21 | 0.13 |
|  | COOP354622 | 0.80 | 0.50 | 0.40 | 0.26 | 0.17 | 0.11 |
|  | COOP355221 | 1.05 | 0.69 | 0.60 | 0.46 | 0.34 | 0.25 |
|  | COOP357631 | 1.19 | 0.83 | 0.73 | 0.54 | 0.38 | 0.26 |
|  | COOP358512 | 0.99 | 0.65 | 0.54 | 0.39 | 0.27 | 0.19 |
| **Region ID** | | **Region 2** | | | | | |
| **Duration** | | **1-hr** | **2-hr** | **3-hr** | **6-hr** | **12-hr** | **24-hr** |
| **Parameters of Fitted Regional GEV Distribution** | Location | 0.80 | 0.85 | 0.86 | 0.86 | 0.85 | 0.84 |
|  | Scale | 0.24 | 0.23 | 0.22 | 0.24 | 0.25 | 0.27 |
|  | Shape | -0.22 | -0.08 | -0.06 | 0.02 | -0.03 | -0.03 |
| **Site-specific Scale Parameter** | COOP103143 | 1.02 | 0.64 | 0.50 | 0.32 | 0.22 | 0.14 |
|  | COOP105241 | 0.72 | 0.45 | 0.37 | 0.22 | 0.13 | 0.08 |
|  | COOP351914 | 1.17 | 0.83 | 0.70 | 0.56 | 0.42 | 0.31 |
|  | COOP352292 | 1.12 | 0.78 | 0.68 | 0.52 | 0.39 | 0.29 |
|  | COOP352295 | 1.15 | 0.80 | 0.70 | 0.47 | 0.32 | 0.20 |
|  | COOP352345 | 1.02 | 0.68 | 0.54 | 0.42 | 0.30 | 0.21 |
|  | COOP352348 | 1.18 | 0.79 | 0.64 | 0.45 | 0.31 | 0.22 |
|  | COOP353305 | 1.12 | 0.79 | 0.67 | 0.49 | 0.32 | 0.22 |
|  | COOP353915 | 0.97 | 0.66 | 0.52 | 0.35 | 0.26 | 0.18 |
|  | COOP355055 | 0.96 | 0.61 | 0.50 | 0.36 | 0.25 | 0.16 |
|  | COOP356546 | 0.75 | 0.48 | 0.38 | 0.25 | 0.15 | 0.09 |
|  | COOP358263 | 1.01 | 0.76 | 0.66 | 0.46 | 0.31 | 0.21 |
|  | COOP358466 | 0.85 | 0.66 | 0.54 | 0.43 | 0.32 | 0.24 |
|  | COOP358790 | 1.23 | 0.80 | 0.65 | 0.46 | 0.35 | 0.24 |
|  | COOP359213 | 0.82 | 0.54 | 0.42 | 0.28 | 0.17 | 0.10 |
|  | COOP451160 | 1.18 | 0.85 | 0.71 | 0.56 | 0.45 | 0.32 |
|  | COOP452030 | 0.68 | 0.47 | 0.38 | 0.27 | 0.18 | 0.11 |
|  | COOP453183 | 1.12 | 0.71 | 0.53 | 0.38 | 0.28 | 0.20 |
| **Region ID** | | **Region 3** | | | | | |
| **Duration** | | **1-hr** | **2-hr** | **3-hr** | **6-hr** | **12-hr** | **24-hr** |
| **Parameters of Fitted Regional GEV Distribution** | Location | 0.75 | 0.79 | 0.82 | 0.82 | 0.84 | 0.83 |
|  | Scale | 0.26 | 0.23 | 0.23 | 0.25 | 0.25 | 0.27 |
|  | Shape | -0.27 | -0.28 | -0.18 | -0.11 | -0.06 | -0.04 |
| **Site-specific Scale Parameter** | COOP105708 | 0.88 | 0.55 | 0.41 | 0.28 | 0.18 | 0.12 |
|  | COOP350694 | 0.95 | 0.55 | 0.42 | 0.26 | 0.18 | 0.12 |
|  | COOP353232 | 0.86 | 0.52 | 0.40 | 0.27 | 0.18 | 0.12 |
|  | COOP353402 | 1.26 | 0.85 | 0.68 | 0.50 | 0.39 | 0.27 |
|  | COOP354321 | 0.75 | 0.47 | 0.35 | 0.22 | 0.14 | 0.09 |
|  | COOP357554 | 1.32 | 0.82 | 0.65 | 0.45 | 0.33 | 0.24 |
| **Region ID** | | **Region 4** | | | | | |
| **Duration** | | **1-hr** | **2-hr** | **3-hr** | **6-hr** | **12-hr** | **24-hr** |
| **Parameters of Fitted Regional GEV Distribution** | Location | 0.82 | 0.86 | 0.88 | 0.87 | 0.85 | 0.84 |
|  | Scale | 0.23 | 0.21 | 0.22 | 0.24 | 0.25 | 0.27 |
|  | Shape | -0.17 | -0.07 | 0.01 | 0.03 | -0.03 | -0.02 |
| **Site-specific Scale Parameter** | COOP350781 | 1.12 | 0.75 | 0.58 | 0.42 | 0.29 | 0.18 |
|  | COOP350897 | 1.28 | 0.83 | 0.68 | 0.51 | 0.37 | 0.25 |
|  | COOP351033 | 1.15 | 0.78 | 0.68 | 0.50 | 0.37 | 0.25 |
|  | COOP351222 | 0.93 | 0.67 | 0.59 | 0.45 | 0.33 | 0.23 |
|  | COOP351643 | 1.04 | 0.69 | 0.58 | 0.43 | 0.31 | 0.21 |
|  | COOP351735 | 1.18 | 0.78 | 0.65 | 0.46 | 0.31 | 0.20 |
|  | COOP351902 | 1.02 | 0.72 | 0.60 | 0.46 | 0.33 | 0.23 |
|  | COOP352374 | 1.23 | 0.75 | 0.60 | 0.41 | 0.28 | 0.18 |
|  | COOP352408 | 1.09 | 0.79 | 0.63 | 0.44 | 0.32 | 0.22 |
|  | COOP353318 | 1.18 | 0.87 | 0.75 | 0.55 | 0.41 | 0.29 |
|  | COOP353340 | 1.05 | 0.69 | 0.55 | 0.37 | 0.26 | 0.18 |
|  | COOP353421 | 1.05 | 0.79 | 0.66 | 0.49 | 0.36 | 0.24 |
|  | COOP353521 | 1.19 | 0.76 | 0.59 | 0.41 | 0.28 | 0.18 |
|  | COOP353705 | 1.09 | 0.79 | 0.67 | 0.53 | 0.37 | 0.26 |
|  | COOP354008 | 0.88 | 0.60 | 0.51 | 0.37 | 0.26 | 0.18 |
|  | COOP354238 | 1.07 | 0.69 | 0.57 | 0.39 | 0.27 | 0.18 |
|  | COOP354276 | 1.13 | 0.83 | 0.73 | 0.55 | 0.42 | 0.29 |
|  | COOP354824 | 1.47 | 1.18 | 1.04 | 0.83 | 0.63 | 0.46 |
|  | COOP355050 | 1.05 | 0.70 | 0.58 | 0.40 | 0.29 | 0.20 |
|  | COOP355206 | 1.45 | 1.03 | 0.84 | 0.64 | 0.46 | 0.32 |
|  | COOP355213 | 0.94 | 0.68 | 0.57 | 0.40 | 0.29 | 0.20 |
|  | COOP355969 | 1.43 | 0.93 | 0.75 | 0.51 | 0.37 | 0.26 |
|  | COOP357127 | 1.02 | 0.66 | 0.57 | 0.39 | 0.29 | 0.19 |
|  | COOP357572 | 1.06 | 0.70 | 0.57 | 0.42 | 0.28 | 0.19 |
|  | COOP357823 | 1.23 | 0.77 | 0.59 | 0.41 | 0.28 | 0.18 |
|  | COOP358182 | 1.42 | 0.93 | 0.72 | 0.51 | 0.36 | 0.24 |
|  | COOP358504 | 1.16 | 0.91 | 0.81 | 0.67 | 0.50 | 0.35 |
|  | COOP358884 | 0.99 | 0.66 | 0.55 | 0.42 | 0.31 | 0.22 |
|  | COOP451759 | 1.29 | 0.91 | 0.82 | 0.63 | 0.47 | 0.33 |

**Table** **S4** Location, scale, and shape parameter of the fitted regional GEV distribution and site-specific scale parameters for the HJA EF region.

| **EFR Name** | **Duration** | **Parameters of Fitted Regional GEV Distribution** | | | **Site-specific Scale Parameter** | | |
| --- | --- | --- | --- | --- | --- | --- | --- |
|  |  | **Location** | **Scale** | **Shape** | **HJA-H15MET** | **HJA-PRIMET** | **HJA-UPLMET** |
| HJA | 15-min | 0.84 | 0.33 | 0.11 | 2.26 | 2.18 | 2.19 |
|  | 30-min | 0.85 | 0.23 | -0.05 | 1.56 | 1.35 | 1.64 |
|  | 1-hr | 0.89 | 0.20 | 0.04 | 1.15 | 0.92 | 1.14 |
|  | 2-hr | 0.92 | 0.21 | 0.26 | 0.88 | 0.71 | 0.95 |
|  | 3-hr | 0.92 | 0.21 | 0.28 | 0.79 | 0.63 | 0.86 |
|  | 6-hr | 0.92 | 0.23 | 0.27 | 0.65 | 0.49 | 0.71 |
|  | 12-hr | 0.90 | 0.26 | 0.25 | 0.52 | 0.41 | 0.55 |
|  | 24-hr | 0.87 | 0.25 | 0.08 | 0.37 | 0.21 | 0.41 |

**Table S5** Estimated, upper and lower bounds (90% Confidence Interval) of Precipitation Intensity (PI) and relative differences (%) between AMS based PIs obtained from Onsite-RFA and NOAA-RFA for HJA-PRIMET station.

| **PIs based on ONSITE-RFA (cm/hr)** | | | | | | | | | | | | |
| --- | --- | --- | --- | --- | --- | --- | --- | --- | --- | --- | --- | --- |
| **Duration** | **25-yr** | | | | **50-yr** | | | | **100-yr** | | | |
|  | **Lower** | **est.** | **upper** | **Rel. Diff. (%)** | **Lower** | **est.** | **upper** | **Rel. Diff. (%)** | **Lower** | **est.** | **upper** | **Rel. Diff. (%)** |
| **15-min** | 3.41 | 3.75 | 4.10 |  | 3.67 | 4.09 | 4.51 |  | 3.88 | 4.39 | 4.92 |  |
| **30-min** | 2.04 | 2.24 | 2.51 |  | 2.25 | 2.51 | 2.88 |  | 2.43 | 2.78 | 3.27 |  |
| **1-hr** | 1.28 | 1.38 | 1.51 | -32.5 | 1.37 | 1.50 | 1.66 | -38.5 | 1.45 | 1.60 | 1.81 | -44.8 |
| **2-hr** | 0.93 | 0.98 | 1.04 | -24.8 | 0.95 | 1.02 | 1.09 | -30.4 | 0.98 | 1.06 | 1.13 | -35.8 |
| **3-hr** | 0.82 | 0.86 | 0.91 | -22.6 | 0.85 | 0.90 | 0.96 | -27.5 | 0.87 | 0.93 | 0.99 | -32.3 |
| **6-hr** | 0.65 | 0.69 | 0.73 | -21.7 | 0.67 | 0.72 | 0.76 | -25.9 | 0.69 | 0.75 | 0.80 | -29.8 |
| **12-hr** | 0.56 | 0.60 | 0.64 | -13.9 | 0.59 | 0.63 | 0.68 | -19.1 | 0.60 | 0.65 | 0.71 | -24.0 |
| **24-hr** | 0.31 | 0.33 | 0.37 | -35.2 | 0.33 | 0.36 | 0.40 | -37.3 | 0.35 | 0.39 | 0.44 | -39.3 |
| **PIs based on NOAA-RFA (cm/hr)** | | | | | | | | | | | |  |
| **1-hr** | 1.79 | 2.05 | 2.43 | - | 2.05 | 2.43 | 3.01 | - | 2.38 | 2.91 | 3.79 | - |
| **2-hr** | 1.17 | 1.30 | 1.47 | - | 1.29 | 1.47 | 1.72 | - | 1.41 | 1.65 | 1.99 | - |
| **3-hr** | 1.02 | 1.12 | 1.24 | - | 1.11 | 1.24 | 1.41 | - | 1.19 | 1.37 | 1.59 | - |
| **6-hr** | 0.81 | 0.88 | 0.98 | - | 0.87 | 0.97 | 1.09 | - | 0.93 | 1.06 | 1.22 | - |
| **12-hr** | 0.63 | 0.70 | 0.78 | - | 0.69 | 0.78 | 0.89 | - | 0.75 | 0.86 | 1.01 | - |
| **24-hr** | 0.47 | 0.52 | 0.58 | - | 0.51 | 0.58 | 0.66 | - | 0.56 | 0.64 | 0.75 | - |

**Table S6** Same as in Table S5 but for HJA-H15MET station.

| **PIs based on ONSITE-RFA (cm/hr)** | | | | | | | | | | | | |
| --- | --- | --- | --- | --- | --- | --- | --- | --- | --- | --- | --- | --- |
| **Duration** | **25-yr** | | | | **50-yr** | | | | **100-yr** | | | |
|  | **Lower** | **est.** | **upper** | **Rel. Diff. (%)** | **Lower** | **est.** | **upper** | **Rel. Diff. (%)** | **Lower** | **est.** | **upper** | **Rel. Diff. (%)** |
| **15-min** | 3.51 | 3.89 | 4.29 | - | 3.78 | 4.23 | 4.72 | - | 4.00 | 4.56 | 5.16 | - |
| **30-min** | 2.35 | 2.59 | 2.89 | - | 2.58 | 2.90 | 3.30 | - | 2.80 | 3.22 | 3.75 | - |
| **1-hr** | 1.59 | 1.72 | 1.87 | -15.6 | 1.69 | 1.86 | 2.05 | -25.3 | 1.78 | 2.00 | 2.23 | -34.6 |
| **2-hr** | 1.13 | 1.20 | 1.28 | 0.92 | 1.17 | 1.25 | 1.34 | -10.1 | 1.20 | 1.29 | 1.39 | -21.2 |
| **3-hr** | 1.02 | 1.08 | 1.14 | 7.8 | 1.05 | 1.13 | 1.20 | -2.94 | 1.08 | 1.16 | 1.24 | -12.8 |
| **6-hr** | 0.86 | 0.92 | 0.98 | 14.6 | 0.90 | 0.96 | 1.02 | 6.07 | 0.92 | 0.99 | 1.06 | -1.76 |
| **12-hr** | 0.71 | 0.76 | 0.81 | 26.4 | 0.74 | 0.80 | 0.86 | 18.3 | 0.76 | 0.83 | 0.89 | 10.6 |
| **24-hr** | 0.55 | 0.59 | 0.66 | 33.4 | 0.58 | 0.65 | 0.72 | 29.6 | 0.62 | 0.69 | 0.78 | 25.9 |
| **PIs based on NOAA-RFA (cm/hr)** | | | | | | | | | | | | |
| **1-hr** | 1.75 | 2.04 | 2.47 | - | 2.07 | 2.49 | 3.17 | - | 2.44 | 3.04 | 4.10 | - |
| **2-hr** | 1.04 | 1.19 | 1.42 | - | 1.19 | 1.39 | 1.72 | - | 1.33 | 1.64 | 2.25 | - |
| **3-hr** | 0.89 | 1.00 | 1.15 | - | 0.99 | 1.16 | 1.37 | - | 1.10 | 1.33 | 1.64 | - |
| **6-hr** | 0.72 | 0.80 | 0.90 | - | 0.79 | 0.90 | 1.04 | - | 0.87 | 1.01 | 1.20 | - |
| **12-hr** | 0.53 | 0.60 | 0.68 | - | 0.59 | 0.67 | 0.77 | - | 0.63 | 0.75 | 0.88 | - |
| **24-hr** | 0.40 | 0.45 | 0.50 | - | 0.44 | 0.50 | 0.57 | - | 0.47 | 0.55 | 0.64 | - |

**Table S7** Same as in Table S5 but for HJA-UPLMET station.

| **PIs based on ONSITE-RFA (cm/hr)** | | | | | | | | | | | | |
| --- | --- | --- | --- | --- | --- | --- | --- | --- | --- | --- | --- | --- |
| **Duration** | **25-yr** | | | | **50-yr** | | | | **100-yr** | | | |
|  | **Lower** | **est.** | **upper** | **Rel. Diff. (%)** | **Lower** | **est.** | **upper** | **Rel. Diff. (%)** | **Lower** | **est.** | **upper** | **Rel. Diff. (%)** |
| **15-min** | 3.38 | 3.77 | 4.23 | - | 3.62 | 4.10 | 4.66 | - | 3.84 | 4.42 | 5.08 | - |
| **30-min** | 2.46 | 2.73 | 3.08 | - | 2.70 | 3.05 | 3.52 | - | 2.92 | 3.38 | 3.99 | - |
| **1-hr** | 1.58 | 1.71 | 1.88 | -16.3 | 1.68 | 1.85 | 2.07 | -24.2 | 1.77 | 1.99 | 2.25 | -32.1 |
| **2-hr** | 1.21 | 1.30 | 1.41 | -1.6 | 1.26 | 1.35 | 1.47 | -8.18 | 1.29 | 1.40 | 1.53 | -12.3 |
| **3-hr** | 1.11 | 1.19 | 1.27 | 5.9 | 1.15 | 1.23 | 1.33 | -1.61 | 1.18 | 1.27 | 1.38 | -8.65 |
| **6-hr** | 0.93 | 0.99 | 1.07 | 13.9 | 0.96 | 1.04 | 1.12 | 6.93 | 0.98 | 1.07 | 1.16 | 0.36 |
| **12-hr** | 0.75 | 0.81 | 0.88 | 20.8 | 0.78 | 0.85 | 0.92 | 13.2 | 0.80 | 0.88 | 0.97 | 6.08 |
| **24-hr** | 0.60 | 0.66 | 0.74 | 34.6 | 0.64 | 0.71 | 0.81 | 30.0 | 0.67 | 0.77 | 0.88 | 25.4 |
| **PIs based on NOAA-RFA (cm/hr)** | | | | | | | | | | | |  |
| **1-hr** | 1.79 | 2.05 | 2.43 | - | 2.06 | 2.45 | 3.04 | - | 2.39 | 2.93 | 3.86 | - |
| **2-hr** | 1.18 | 1.32 | 1.48 | - | 1.31 | 1.47 | 1.66 | - | 1.41 | 1.60 | 2.06 | - |
| **3-hr** | 1.01 | 1.12 | 1.25 | - | 1.11 | 1.26 | 1.43 | - | 1.20 | 1.40 | 1.63 | - |
| **6-hr** | 0.79 | 0.87 | 0.97 | - | 0.86 | 0.97 | 1.10 | - | 0.93 | 1.07 | 1.24 | - |
| **12-hr** | 0.60 | 0.67 | 0.75 | - | 0.66 | 0.75 | 0.86 | - | 0.72 | 0.83 | 0.98 | - |
| **24-hr** | 0.44 | 0.49 | 0.55 | - | 0.49 | 0.55 | 0.63 | - | 0.53 | 0.61 | 0.72 | - |

**Table S8** Location, scale, shape parameter of the fitted regional GEV distribution and site-specific scale parameters for the CHL EF region.

| **EFR Name** | **Duration** | **Parameters of Fitted Regional GEV Distribution** | | | **Site-specific Scale Parameter** | | |
| --- | --- | --- | --- | --- | --- | --- | --- |
|  |  | **Location** | **Scale** | **Shape** | **CHL-RRG31** | **CHL-RRG06** | **CHL-RRG41** |
| **CHL** | 1-hr | 0.83 | 0.22 | -0.15 | 3.67 | 3.40 | 3.40 |
|  | 2-hr | 0.84 | 0.24 | -0.06 | 2.55 | 2.30 | 2.31 |
|  | 3-hr | 0.86 | 0.23 | -0.03 | 2.04 | 1.79 | 1.80 |
|  | 6-hr | 0.89 | 0.21 | 0.08 | 1.40 | 1.18 | 1.19 |
|  | 12-hr | 0.89 | 0.23 | 0.12 | 0.98 | 0.80 | 0.81 |
|  | 24-hr | 0.88 | 0.24 | 0.10 | 0.63 | 0.50 | 0.50 |

**Table S9** Same as in Table S5 but PIs obtained from Onsite-RFA and NOAA-Atlas14 for CHL-RRG06 station.

| **PIs based on ONSITE-RFA (cm/hr)** | | | | | | | | | | | | |
| --- | --- | --- | --- | --- | --- | --- | --- | --- | --- | --- | --- | --- |
| **Duration** | **25-yr** | | | | **50-yr** | | | | **100-yr** | | | |
|  | **Lower** | **est.** | **upper** | **Rel. Diff. (%)** | **Lower** | **est.** | **upper** | **Rel. Diff. (%)** | **Lower** | **est.** | **upper** | **Rel. Diff. (%)** |
| **1-hr** | 5.33 | 5.94 | 6.69 | -18.0 | 5.99 | 6.85 | 7.95 | -17.5 | 6.67 | 7.86 | 9.45 | -16.4 |
| **2-hr** | 3.52 | 3.91 | 4.37 | -7.89 | 3.87 | 4.39 | 5.02 | -9.95 | 4.21 | 4.89 | 5.72 | -11.2 |
| **3-hr** | 2.66 | 2.93 | 3.25 | -4.70 | 2.90 | 3.25 | 3.69 | -8.47 | 3.11 | 3.59 | 4.16 | -11.7 |
| **6-hr** | 1.64 | 1.76 | 1.91 | -10.2 | 1.73 | 1.89 | 2.09 | -16.5 | 1.81 | 2.01 | 2.25 | -21.6 |
| **12-hr** | 1.13 | 1.21 | 1.31 | -3.46 | 1.19 | 1.30 | 1.43 | -9.11 | 1.24 | 1.37 | 1.54 | -13.7 |
| **24-hr** | 0.72 | 0.77 | 0.84 | -6.43 | 0.76 | 0.83 | 0.92 | -10.4 | 0.79 | 0.89 | 1.00 | -14.2 |
| **PIs based on NOAA-Atlas14 (cm/hr)** | | | | | | | | | | | | |
| **1-hr** | 6.45 | 7.24 | 8.00 | - | 7.39 | 8.31 | 9.22 | - | 8.28 | 9.40 | 10.4 | - |
| **2-hr** | 3.81 | 4.24 | 4.67 | - | 4.34 | 4.88 | 5.38 | - | 4.88 | 5.51 | 6.12 | - |
| **3-hr** | 2.77 | 3.07 | 3.38 | - | 3.20 | 3.56 | 3.94 | - | 3.61 | 4.06 | 4.50 | - |
| **6-hr** | 1.77 | 1.96 | 2.15 | - | 2.03 | 2.26 | 2.49 | - | 2.28 | 2.57 | 2.84 | - |
| **12-hr** | 1.14 | 1.25 | 1.37 | - | 1.28 | 1.42 | 1.57 | - | 1.42 | 1.59 | 1.76 | - |
| **24-hr** | 0.76 | 0.83 | 0.89 | - | 0.85 | 0.93 | 1.01 | - | 0.94 | 1.04 | 1.13 | - |

**Table S10** Same as in Table S5 but PIs obtained from Onsite-RFA and NOAA-Atlas14 for CHL-RRG41 station.

| **PIs based on ONSITE-RFA (cm/hr)** | | | | | | | | | | | | |
| --- | --- | --- | --- | --- | --- | --- | --- | --- | --- | --- | --- | --- |
| **Duration** | **25-yr** | | | | **50yr** | | | | **100-yr** | | | |
|  | **Lower** | **est.** | **upper** | **Rel. Diff. (%)** | **Lower** | **est.** | **upper** | **Rel. Diff. (%)** | **Lower** | **est.** | **upper** | **Rel. Diff. (%)** |
| **1-hr** | 5.30 | 5.93 | 6.67 | -18.3 | 5.96 | 6.85 | 7.93 | -18.0 | 6.64 | 7.86 | 9.37 | -16.6 |
| **2-hr** | 3.56 | 3.93 | 4.37 | -8.01 | 3.90 | 4.41 | 5.02 | -10.0 | 4.22 | 4.91 | 5.74 | -11.2 |
| **3-hr** | 2.67 | 2.94 | 3.27 | -5.17 | 2.91 | 3.27 | 3.71 | -8.82 | 3.12 | 3.60 | 4.20 | -11.5 |
| **6-hr** | 1.65 | 1.78 | 1.92 | -9.71 | 1.74 | 1.91 | 2.10 | -16.0 | 1.82 | 2.03 | 2.27 | -21.5 |
| **12-hr** | 1.13 | 1.21 | 1.32 | -3.77 | 1.19 | 1.30 | 1.44 | -9.31 | 1.24 | 1.38 | 1.55 | -14.0 |
| **24-hr** | 0.71 | 0.77 | 0.84 | -7.16 | 0.75 | 0.83 | 0.92 | -11.1 | 0.78 | 0.89 | 1.01 | -14.8 |
| **PIs based on NOAA-Atlas14 (cm/hr)** | | | | | | | | | | | | |
| **1-hr** | 6.48 | 7.26 | 8.03 | - | 7.42 | 8.36 | 9.27 | - | 8.31 | 9.42 | 10.5 | - |
| **2-hr** | 3.84 | 4.27 | 4.70 | - | 4.37 | 4.90 | 5.41 | - | 4.90 | 5.54 | 6.15 | - |
| **3-hr** | 2.79 | 3.10 | 3.40 | - | 3.20 | 3.58 | 3.94 | - | 3.61 | 4.06 | 4.52 | - |
| **6-hr** | 1.78 | 1.97 | 2.16 | - | 2.04 | 2.27 | 2.51 | - | 2.30 | 2.59 | 2.87 | - |
| **12-hr** | 1.14 | 1.26 | 1.38 | - | 1.29 | 1.44 | 1.58 | - | 1.43 | 1.61 | 1.77 | - |
| **24-hr** | 0.76 | 0.83 | 0.90 | - | 0.86 | 0.93 | 1.01 | - | 0.95 | 1.04 | 1.13 | - |

**Table S11** Same as in Table S5 but PIs obtained from Onsite-RFA and NOAA-Atlas14 for CHL-RRG31 station.

| **PIs based on ONSITE-RFA (cm/hr)** | | | | | | | | | | | | |
| --- | --- | --- | --- | --- | --- | --- | --- | --- | --- | --- | --- | --- |
| **Duration** | **25-yr** | | | | **50-yr** | | | | **100-yr** | | | |
|  | **Lower** | **est.** | **upper** | **Rel. Diff. (%)** | **Lower** | **est.** | **upper** | **Rel. Diff. (%)** | **Lower** | **est.** | **upper** | **Rel. Diff. (%)** |
| **1-hr** | 5.76 | 6.41 | 7.23 | -18.1 | 6.51 | 7.40 | 8.54 | -18.0 | 7.26 | 8.49 | 10.1 | -16.7 |
| **2-hr** | 3.94 | 4.33 | 4.83 | -3.61 | 4.33 | 4.87 | 5.57 | -6.00 | 4.69 | 5.43 | 6.36 | -7.52 |
| **3-hr** | 3.06 | 3.34 | 3.72 | 1.88 | 3.33 | 3.71 | 4.23 | -2.63 | 3.58 | 4.09 | 4.78 | -5.89 |
| **6-hr** | 1.94 | 2.09 | 2.26 | -1.51 | 2.06 | 2.24 | 2.47 | -8.63 | 2.15 | 2.39 | 2.67 | -14.6 |
| **12-hr** | 1.37 | 1.48 | 1.61 | 6.12 | 1.45 | 1.58 | 1.75 | -0.16 | 1.51 | 1.68 | 1.89 | -5.45 |
| **24-hr** | 0.90 | 0.98 | 1.07 | 3.64 | 0.95 | 1.05 | 1.17 | -0.81 | 1.00 | 1.12 | 1.27 | -5.25 |
| **PIs based on NOAA-Atlas14 (cm/hr)** | | | | | | | | | | | | |
| **1-hr** | 6.99 | 7.82 | 8.66 | - | 8.00 | 9.02 | 10.0 | - | 8.97 | 10.2 | 11.4 | - |
| **2-hr** | 4.04 | 4.50 | 4.98 | - | 4.62 | 5.18 | 5.74 | - | 5.18 | 5.87 | 6.50 | - |
| **3-hr** | 2.95 | 3.28 | 3.61 | - | 3.40 | 3.81 | 4.22 | - | 3.84 | 4.34 | 4.80 | - |
| **6-hr** | 1.91 | 2.12 | 2.34 | - | 2.19 | 2.45 | 2.72 | - | 2.47 | 2.79 | 3.12 | - |
| **12-hr** | 1.26 | 1.39 | 1.53 | - | 1.42 | 1.59 | 1.75 | - | 1.58 | 1.78 | 1.97 | - |
| **24-hr** | 0.87 | 0.94 | 1.02 | - | 0.97 | 1.06 | 1.16 | - | 1.07 | 1.19 | 1.30 | - |

**Table S12** Location, scale, shape parameter of the fitted GEV distribution for the SAN-MET25 station.

| **Duration** | **Location** | | | **Scale** | | | **Shape** | | |
| --- | --- | --- | --- | --- | --- | --- | --- | --- | --- |
|  | **5%** | **Estimate** | **95%** | **5%** | **Estimate** | **95%** | **5%** | **Estimate** | **95%** |
| **1-hr** | 3.80 | 4.12 | 4.46 | 0.93 | 1.17 | 1.43 | -0.21 | -0.01 | 0.18 |
| **2-hr** | 2.31 | 2.48 | 2.67 | 0.52 | 0.68 | 0.83 | -0.05 | 0.20 | 0.40 |
| **3-hr** | 1.77 | 1.90 | 2.05 | 0.39 | 0.52 | 0.65 | -0.02 | 0.23 | 0.41 |
| **6-hr** | 0.97 | 1.06 | 1.16 | 0.24 | 0.33 | 0.42 | 0.06 | 0.34 | 0.53 |
| **12-hr** | 0.56 | 0.62 | 0.68 | 0.16 | 0.21 | 0.27 | -0.02 | 0.23 | 0.45 |
| **24-hr** | 0.33 | 0.36 | 0.40 | 0.10 | 0.13 | 0.16 | 0.02 | 0.25 | 0.46 |

**Table S13** Same as in Table S5 but PIs obtained from Onsite-LMOM and NOAA-Atlas14 for SAN-MET25 station.

| **PIs based on ONSITE-LMOM (cm/hr)** | | | | | | | | | | | | |
| --- | --- | --- | --- | --- | --- | --- | --- | --- | --- | --- | --- | --- |
| **Duration** | **25-yr** | | | | **50-yr** | | | | **100-yr** | | | |
|  | **Lower** | **est.** | **upper** | **Rel. Diff. (%)** | **Lower** | **est.** | **upper** | **Rel. Diff. (%)** | **Lower** | **est.** | **upper** | **Rel. Diff. (%)** |
| **1-hr** | 6.77 | 7.81 | 8.97 | -6.49 | 7.23 | 8.61 | 10.3 | -9.34 | 7.60 | 9.40 | 11.7 | -11.3 |
| **2-hr** | 4.44 | 5.49 | 6.78 | 3.98 | 4.89 | 6.45 | 8.57 | 6.71 | 5.32 | 7.54 | 10.9 | 10.8 |
| **3-hr** | 3.43 | 4.34 | 5.42 | 13.3 | 3.79 | 5.17 | 7.00 | 16.3 | 4.14 | 6.13 | 9.05 | 21.4 |
| **6-hr** | 2.16 | 2.93 | 3.86 | 27.6 | 2.46 | 3.68 | 5.33 | 38.0 | 2.78 | 4.63 | 7.48 | 51.9 |
| **12-hr** | 1.25 | 1.63 | 2.08 | 20.5 | 1.40 | 1.97 | 2.72 | 24.6 | 1.55 | 2.36 | 3.58 | 30.7 |
| **24-hr** | 0.75 | 0.98 | 1.26 | 20.3 | 0.84 | 1.19 | 1.67 | 27.5 | 0.93 | 1.44 | 2.22 | 36.0 |
| **PIs based on NOAA-Atlas14 (cm/hr)** | | | | | | | | | | | | |
| **1-hr** | 7.72 | 8.36 | 8.99 | - | 8.74 | 9.50 | 10.2 | - | 9.68 | 10.6 | 11.4 | - |
| **2-hr** | 4.85 | 5.28 | 5.66 | - | 5.54 | 6.05 | 6.50 | - | 6.17 | 6.81 | 7.32 | - |
| **3-hr** | 3.51 | 3.84 | 4.17 | - | 4.04 | 4.45 | 4.83 | - | 4.55 | 5.05 | 5.49 | - |
| **6-hr** | 2.09 | 2.29 | 2.49 | - | 2.41 | 2.67 | 2.90 | - | 2.72 | 3.05 | 3.30 | - |
| **12-hr** | 1.22 | 1.35 | 1.47 | - | 1.42 | 1.58 | 1.72 | - | 1.61 | 1.81 | 1.97 | - |
| **24-hr** | 0.74 | 0.81 | 0.89 | - | 0.85 | 0.93 | 1.02 | - | 0.96 | 1.06 | 1.16 | - |

**Table S14** Location, scale, shape parameter of the fitted GEV distribution for the ALC-AC04 station.

| **Duration** | **Location** | | | **Scale** | | | **Shape** | | |
| --- | --- | --- | --- | --- | --- | --- | --- | --- | --- |
|  | **5%** | **Estimate** | **95%** | **5%** | **Estimate** | **95%** | **5%** | **Estimate** | **95%** |
| **1-hr** | 2.61 | 2.92 | 3.30 | 0.92 | 1.20 | 1.46 | -0.31 | -0.09 | 0.09 |
| **2-hr** | 1.59 | 1.81 | 2.07 | 0.61 | 0.77 | 0.94 | -0.30 | -0.08 | 0.09 |
| **3-hr** | 1.19 | 1.34 | 1.52 | 0.44 | 0.57 | 0.69 | -0.24 | -0.02 | 0.17 |
| **6-hr** | 0.70 | 0.79 | 0.90 | 0.27 | 0.34 | 0.42 | -0.20 | 0.03 | 0.22 |
| **12-hr** | 0.43 | 0.49 | 0.55 | 0.15 | 0.20 | 0.24 | -0.23 | 0.00 | 0.19 |
| **24-hr** | 0.24 | 0.27 | 0.31 | 0.09 | 0.12 | 0.15 | -0.16 | 0.07 | 0.27 |

**Table S15** Same as in Table S5 but PIs obtained from Onsite-LMOM and NOAA-Atlas14 for ALC-AC04 station.

| **PIs based on ONSITE-LMOM (cm/hr)** | | | | | | | | | | | | |
| --- | --- | --- | --- | --- | --- | --- | --- | --- | --- | --- | --- | --- |
|  | **25-yr** | | | | **50-yr** | | | | **100-yr** | | | |
| **Duration** | **Lower** | **est.** | **upper** | **Rel. Diff. (%)** | **Lower** | **est.** | **upper** | **Rel. Diff. (%)** | **Lower** | **est.** | **upper** | **Rel. Diff. (%)** |
| **1-hr** | 5.33 | 6.24 | 7.21 | -14.5 | 5.69 | 6.84 | 8.19 | -15.0 | 5.97 | 7.41 | 9.25 | -15.9 |
| **2-hr** | 3.40 | 3.99 | 4.61 | -13.3 | 3.64 | 4.40 | 5.29 | -13.5 | 3.83 | 4.78 | 6.01 | -14.1 |
| **3-hr** | 2.58 | 3.11 | 3.69 | -11.3 | 2.79 | 3.49 | 4.32 | -10.3 | 2.96 | 3.86 | 5.02 | -10.1 |
| **6-hr** | 1.58 | 1.93 | 2.33 | -12.0 | 1.72 | 2.20 | 2.78 | -10.8 | 1.83 | 2.46 | 3.31 | -10.3 |
| **12-hr** | 0.93 | 1.13 | 1.34 | -16.4 | 1.01 | 1.27 | 1.58 | -16.9 | 1.07 | 1.41 | 1.85 | -17.6 |
| **24-hr** | 0.56 | 0.70 | 0.86 | -13.1 | 0.61 | 0.81 | 1.05 | -11.8 | 0.66 | 0.93 | 1.28 | -10.7 |
| **PIs based on NOAA-Atlas14 (cm/hr)** | | | | | | | | | | | | |
| **1-hr** | 6.02 | 7.29 | 8.71 | - | 6.50 | 8.05 | 9.78 | - | 6.88 | 8.81 | 11.0 | - |
| **2-hr** | 3.81 | 4.60 | 5.46 | - | 4.14 | 5.08 | 6.12 | - | 4.37 | 5.56 | 6.88 | - |
| **3-hr** | 2.92 | 3.51 | 4.14 | - | 3.18 | 3.89 | 4.70 | - | 3.38 | 4.29 | 5.28 | - |
| **6-hr** | 1.84 | 2.19 | 2.59 | - | 2.02 | 2.46 | 2.95 | - | 2.17 | 2.74 | 3.35 | - |
| **12-hr** | 1.14 | 1.35 | 1.58 | - | 1.26 | 1.52 | 1.82 | - | 1.37 | 1.71 | 2.09 | - |
| **24-hr** | 0.69 | 0.81 | 0.95 | - | 0.77 | 0.92 | 1.09 | - | 0.84 | 1.04 | 1.26 | - |

**Table S16** Location, scale, shape parameter of the fitted GEV distribution for the FRS-HQTRS station.

| **Duration** | **Location** | | | **Scale** | | | **Shape** | | |
| --- | --- | --- | --- | --- | --- | --- | --- | --- | --- |
|  | **5%** | **Estimate** | **95%** | **5%** | **Estimate** | **95%** | **5%** | **Estimate** | **95%** |
| **1-hr** | 0.74 | 0.83 | 0.96 | 0.15 | 0.24 | 0.37 | -0.09 | 0.40 | 0.66 |
| **2-hr** | 0.47 | 0.53 | 0.61 | 0.10 | 0.16 | 0.23 | -0.15 | 0.26 | 0.56 |
| **3-hr** | 0.33 | 0.38 | 0.43 | 0.06 | 0.10 | 0.16 | -0.15 | 0.29 | 0.54 |
| **6-hr** | 0.23 | 0.25 | 0.28 | 0.04 | 0.06 | 0.08 | -0.18 | 0.24 | 0.52 |
| **12-hr** | 0.13 | 0.15 | 0.17 | 0.03 | 0.04 | 0.05 | -0.34 | 0.01 | 0.27 |
| **24-hr** | 0.08 | 0.09 | 0.10 | 0.02 | 0.02 | 0.03 | -0.37 | 0.00 | 0.32 |

**Table S17** Same as in Table S5 but PIs obtained from Onsite-LMOM and NOAA-Atlas14 for FRS-HQTRS station.

| **PIs based on ONSITE-LMOM (cm/hr)** | | | | | | | | | | | | |
| --- | --- | --- | --- | --- | --- | --- | --- | --- | --- | --- | --- | --- |
|  | **25-yr** | | | | **50-yr** | | | | **100-yr** | | | |
| **Duration** | **Lower** | **est.** | **upper** | **Rel. Diff. (%)** | **Lower** | **est.** | **upper** | **Rel. Diff. (%)** | **Lower** | **est.** | **upper** | **Rel. Diff. (%)** |
| **1-hr** | 1.44 | 2.39 | 3.61 | -38.8 | 1.59 | 3.09 | 5.26 | -32.7 | 1.73 | 4.02 | 7.88 | -24.7 |
| **2-hr** | 0.90 | 1.33 | 1.88 | -42.4 | 0.97 | 1.62 | 2.56 | -40.0 | 1.04 | 1.96 | 3.54 | -36.9 |
| **3-hr** | 0.62 | 0.92 | 1.31 | -41.5 | 0.67 | 1.13 | 1.80 | -38.7 | 0.71 | 1.38 | 2.55 | -34.5 |
| **6-hr** | 0.38 | 0.53 | 0.72 | -35.9 | 0.41 | 0.62 | 0.94 | -34.8 | 0.43 | 0.74 | 1.25 | -32.4 |
| **12-hr** | 0.22 | 0.28 | 0.34 | -34.7 | 0.23 | 0.31 | 0.41 | -37.2 | 0.24 | 0.34 | 0.49 | -39.5 |
| **24-hr** | 0.13 | 0.16 | 0.21 | -28.6 | 0.14 | 0.18 | 0.24 | -31.1 | 0.14 | 0.20 | 0.29 | -33.6 |
| **PIs based on NOAA-Atlas14 (cm/hr)** | | | | | | | | | | | | |
| **1-hr** | 2.92 | 3.91 | 5.31 | - | 3.38 | 4.60 | 6.35 | - | 3.76 | 5.33 | 7.54 | - |
| **2-hr** | 1.73 | 2.31 | 3.07 | - | 1.99 | 2.69 | 3.66 | - | 2.20 | 3.10 | 4.32 | - |
| **3-hr** | 1.19 | 1.58 | 2.09 | - | 1.36 | 1.84 | 2.48 | - | 1.50 | 2.10 | 2.90 | - |
| **6-hr** | 0.63 | 0.82 | 1.08 | - | 0.71 | 0.96 | 1.27 | - | 0.79 | 1.09 | 1.48 | - |
| **12-hr** | 0.33 | 0.43 | 0.56 | - | 0.37 | 0.49 | 0.65 | - | 0.41 | 0.56 | 0.75 | - |
| **24-hr** | 0.18 | 0.23 | 0.30 | - | 0.20 | 0.26 | 0.35 | - | 0.22 | 0.30 | 0.40 | - |

**Table S18** Location, scale, shape parameter of the fitted GEV distribution for the HBR-RG01 site location.

| **Duration** | **Location** | | | **Scale** | | | **Shape** | | |
| --- | --- | --- | --- | --- | --- | --- | --- | --- | --- |
|  | **5%** | **Estimate** | **95%** | **5%** | **Estimate** | **95%** | **5%** | **Estimate** | **95%** |
| **15-min** | 3.83 | 4.16 | 4.53 | 0.98 | 1.24 | 1.53 | -0.18 | 0.02 | 0.19 |
| **30-min** | 2.73 | 2.93 | 3.15 | 0.60 | 0.77 | 0.96 | -0.06 | 0.18 | 0.38 |
| **1-hrs** | 1.75 | 1.90 | 2.08 | 0.44 | 0.57 | 0.73 | -0.05 | 0.18 | 0.39 |
| **2-hrs** | 1.18 | 1.27 | 1.39 | 0.28 | 0.37 | 0.45 | -0.10 | 0.13 | 0.32 |
| **3-hrs** | 0.92 | 0.97 | 1.04 | 0.17 | 0.22 | 0.28 | -0.01 | 0.23 | 0.45 |
| **6-hrs** | 0.62 | 0.68 | 0.73 | 0.15 | 0.19 | 0.24 | -0.21 | 0.00 | 0.16 |
| **12-hrs** | 0.41 | 0.44 | 0.47 | 0.08 | 0.11 | 0.13 | -0.21 | 0.00 | 0.18 |
| **24-hrs** | 0.25 | 0.27 | 0.29 | 0.05 | 0.07 | 0.08 | -0.19 | 0.04 | 0.22 |

**Table S19** Same as in Table S5 but PIs obtained from Onsite-LMOM and NOAA-Atlas14 for HBR-RG01 station.

| **PIs based on ONSITE-LMOM (cm/hr)** | | | | | | | | | | | | |
| --- | --- | --- | --- | --- | --- | --- | --- | --- | --- | --- | --- | --- |
|  | **25-yr** | | | | **50-yr** | | | | **100-yr** | | | |
| **Duration** | **Lower** | **est.** | **upper** | **Rel. Diff. (%)** | **Lower** | **est.** | **upper** | **Rel. Diff. (%)** | **Lower** | **est.** | **upper** | **Rel. Diff. (%)** |
| **15-min** | 7.06 | 8.25 | 9.60 | -20.2 | 7.57 | 9.18 | 11.2 | -21.5 | 7.99 | 10.1 | 12.9 | -22.7 |
| **30-min** | 5.12 | 6.29 | 7.68 | -12.2 | 5.62 | 7.32 | 9.59 | -9.60 | 6.08 | 8.49 | 12.1 | -6.34 |
| **1-hr** | 3.52 | 4.39 | 5.43 | -4.02 | 3.89 | 5.16 | 6.86 | -0.49 | 4.22 | 6.02 | 8.71 | 3.54 |
| **2-hr** | 2.24 | 2.73 | 3.28 | -5.72 | 2.45 | 3.14 | 4.00 | -4.12 | 2.64 | 3.59 | 4.90 | -2.54 |
| **3-hr** | 1.63 | 2.02 | 2.49 | -8.66 | 1.79 | 2.37 | 3.16 | -5.42 | 1.94 | 2.78 | 4.06 | -1.39 |
| **6-hr** | 1.12 | 1.29 | 1.49 | -8.35 | 1.19 | 1.43 | 1.71 | -10.7 | 1.25 | 1.56 | 1.97 | -13.3 |
| **12-hr** | 0.69 | 0.79 | 0.89 | -12.3 | 0.73 | 0.86 | 1.02 | -14.7 | 0.76 | 0.94 | 1.16 | -17.3 |
| **24-hr** | 0.43 | 0.49 | 0.57 | -10.8 | 0.45 | 0.55 | 0.66 | -12.2 | 0.48 | 0.60 | 0.77 | -13.5 |
| **PIs based on NOAA-Atlas14 (cm/hr)** | | | | | | | | | | | | |
| **15-min** | 7.87 | 10.3 | 13.3 | - | 8.69 | 11.7 | 15.3 | - | 9.42 | 13.1 | 17.6 | - |
| **30-min** | 5.44 | 7.16 | 9.17 | - | 6.02 | 8.10 | 10.6 | - | 6.53 | 9.07 | 12.2 | - |
| **1-hr** | 3.48 | 4.57 | 5.87 | - | 3.84 | 5.18 | 6.76 | - | 4.17 | 5.82 | 7.80 | - |
| **2-hr** | 2.21 | 2.90 | 3.68 | - | 2.46 | 3.28 | 4.27 | - | 2.69 | 3.68 | 4.95 | - |
| **3-hr** | 1.70 | 2.21 | 2.82 | - | 1.89 | 2.50 | 3.25 | - | 2.07 | 2.82 | 3.78 | - |
| **6-hr** | 1.09 | 1.41 | 1.79 | - | 1.22 | 1.60 | 2.07 | - | 1.33 | 1.80 | 2.41 | - |
| **12-hr** | 0.70 | 0.90 | 1.12 | - | 0.77 | 1.01 | 1.29 | - | 0.84 | 1.14 | 1.50 | - |
| **24-hr** | 0.43 | 0.55 | 0.69 | - | 0.48 | 0.62 | 0.79 | - | 0.52 | 0.70 | 0.92 | - |

**Figures**


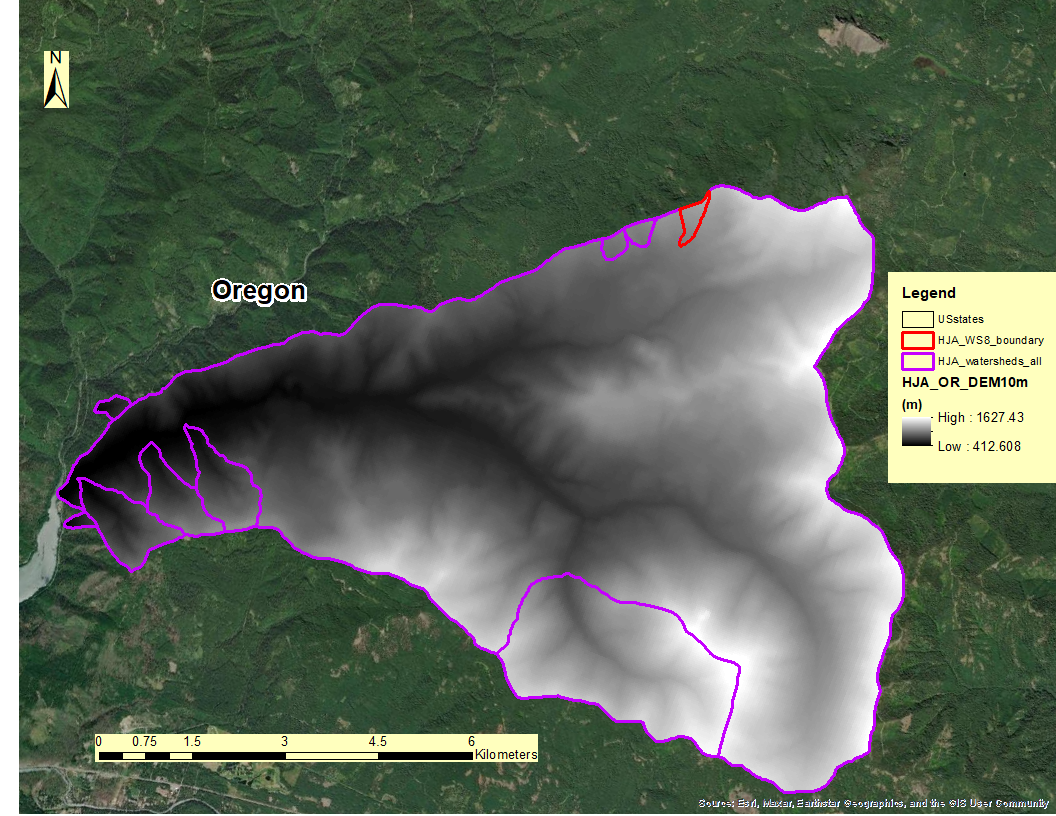


**Fig. S1** DEM Model data for the HJA EF’s watersheds selected in this study.


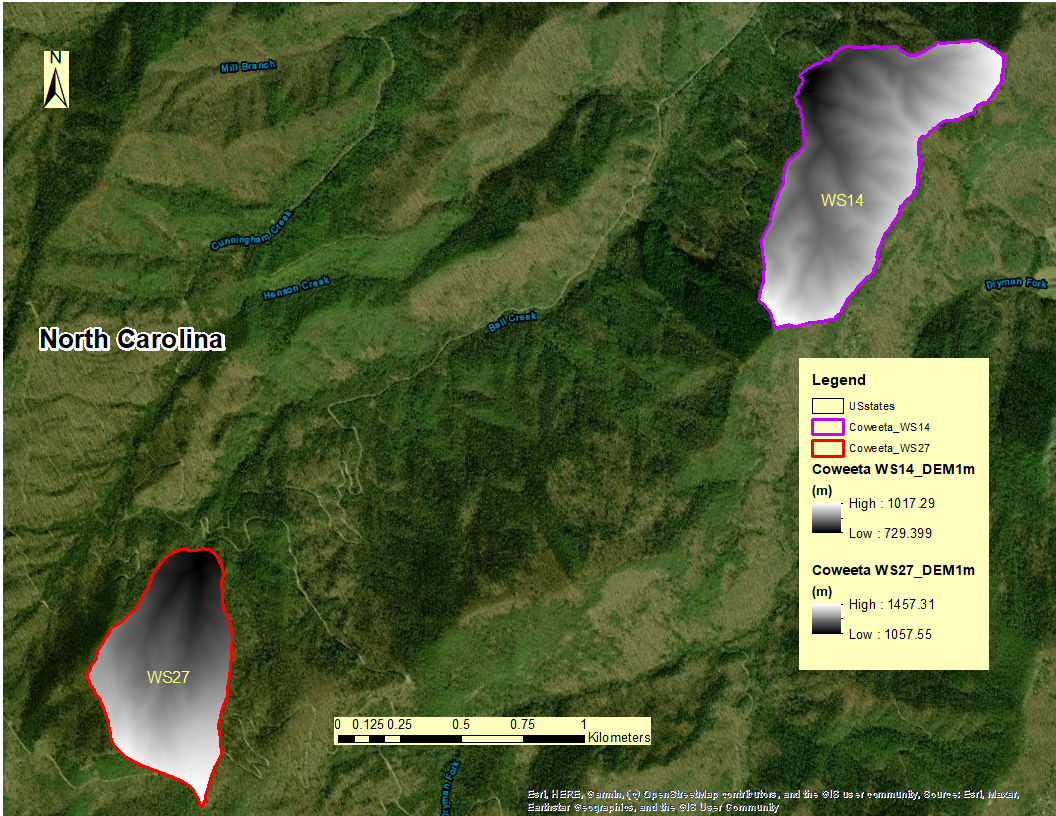


Fig S2 DEM Model data for the CHL EF’s watersheds selected in this study.


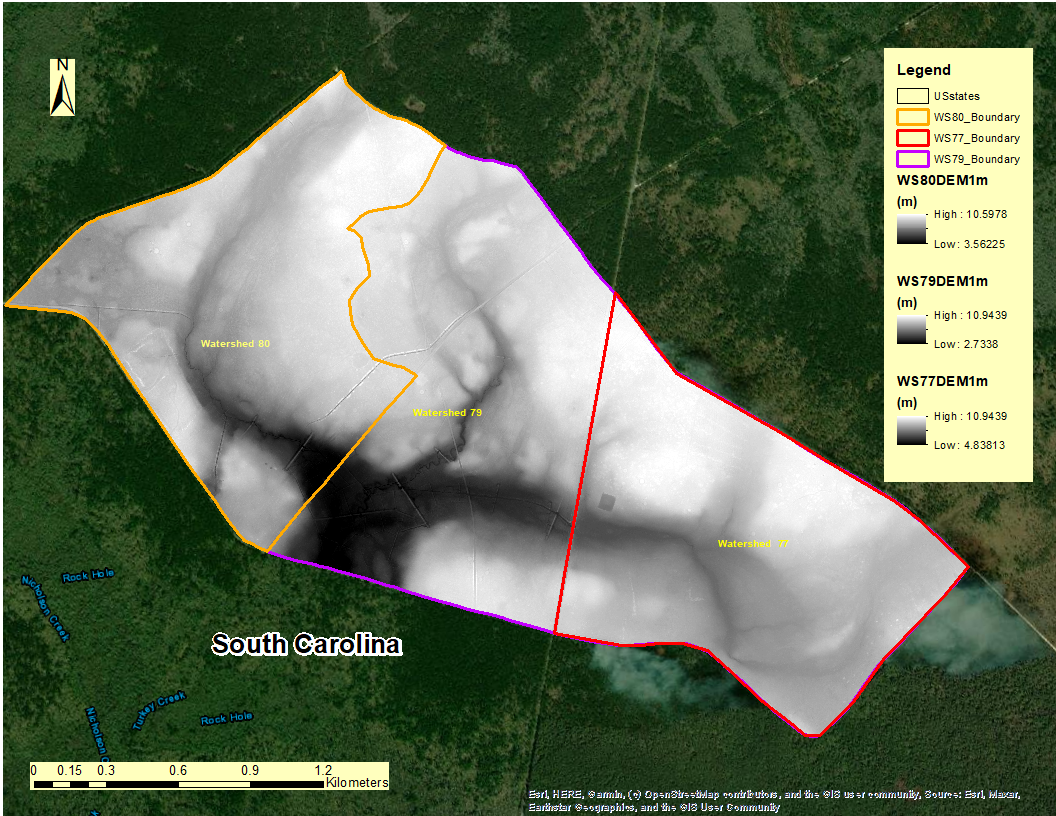


Fig S3 DEM Model data for the SAN EF’s watersheds selected in this study.


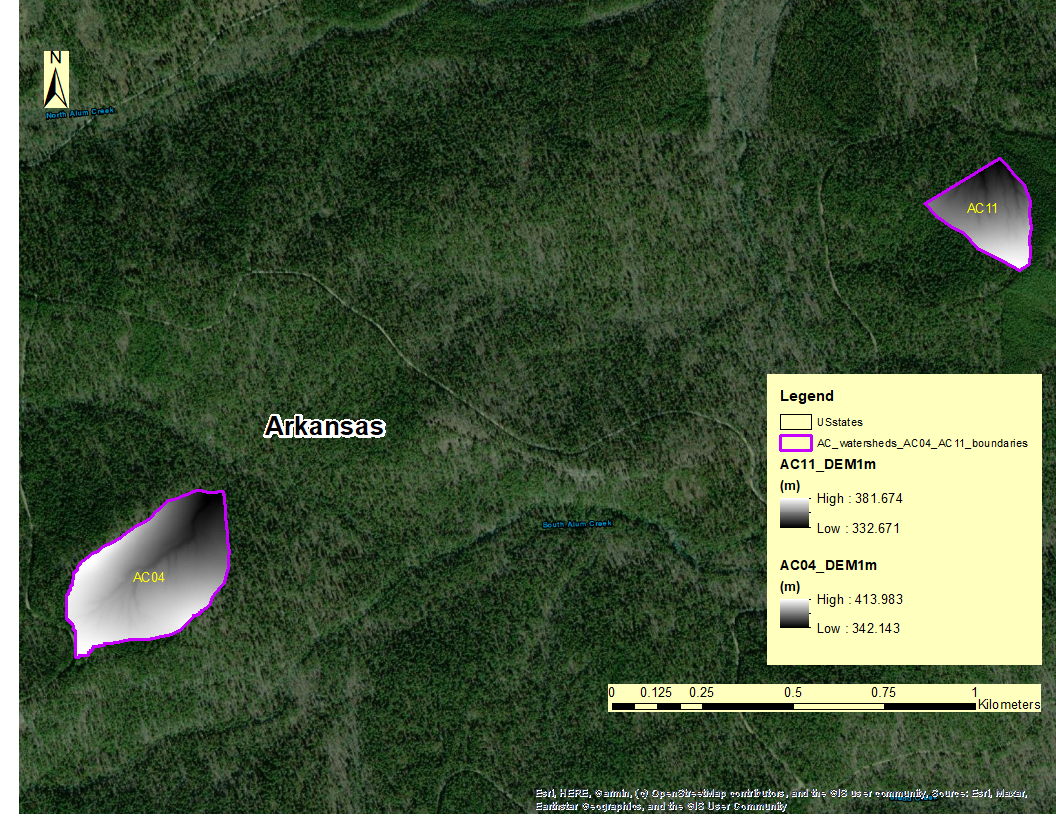


Fig S4 DEM Model data for the ALC EF’s watersheds selected in this study.


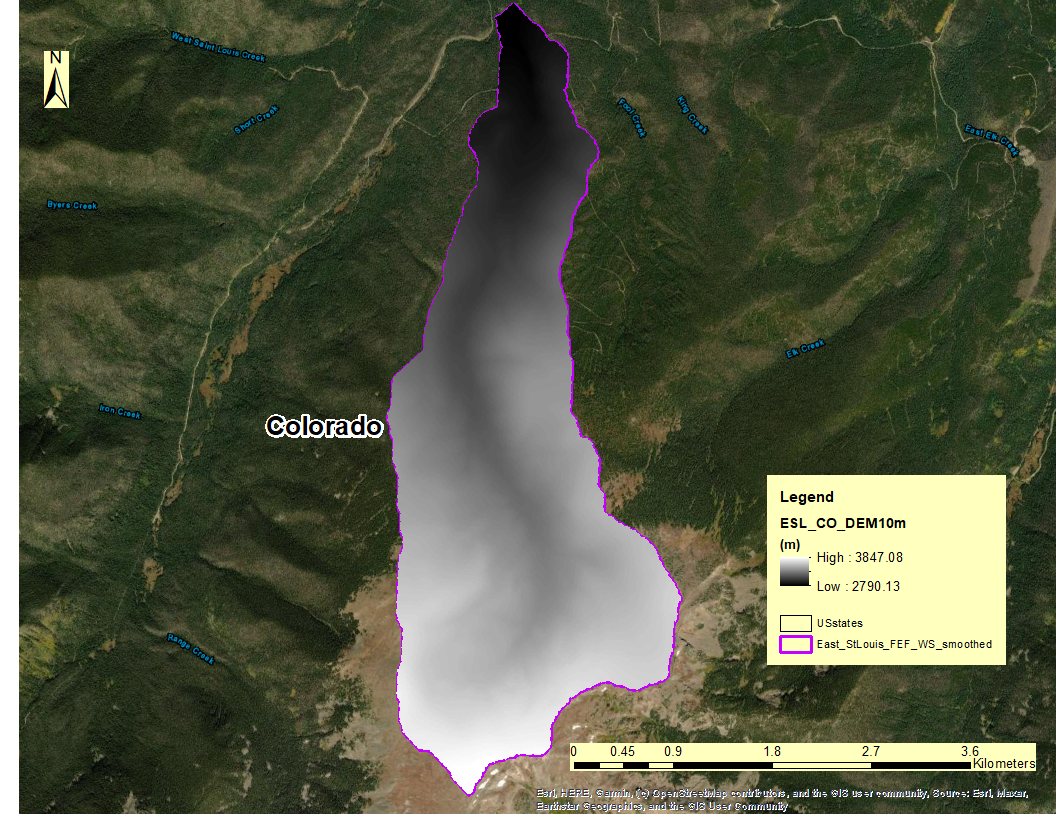


Fig S5 DEM Model data for the FRS EF’s watersheds selected in this study.


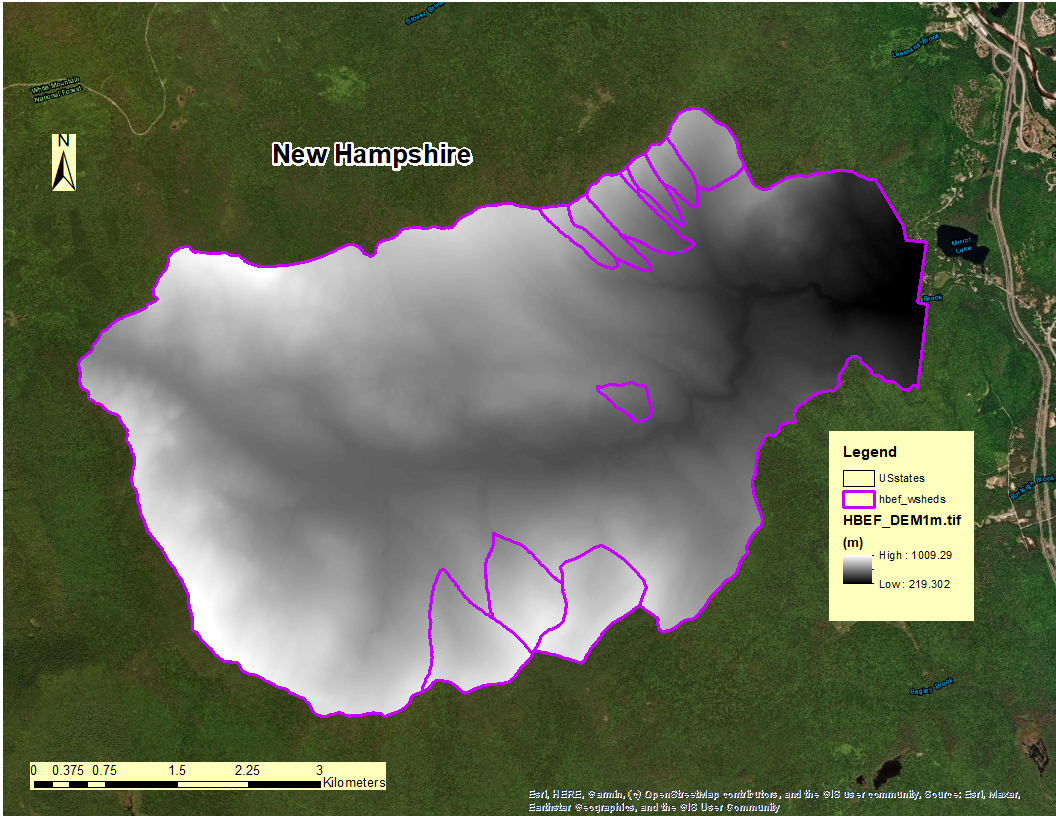


Fig S6 DEM Model data for the HBR EF’s watersheds selected in this study.

**
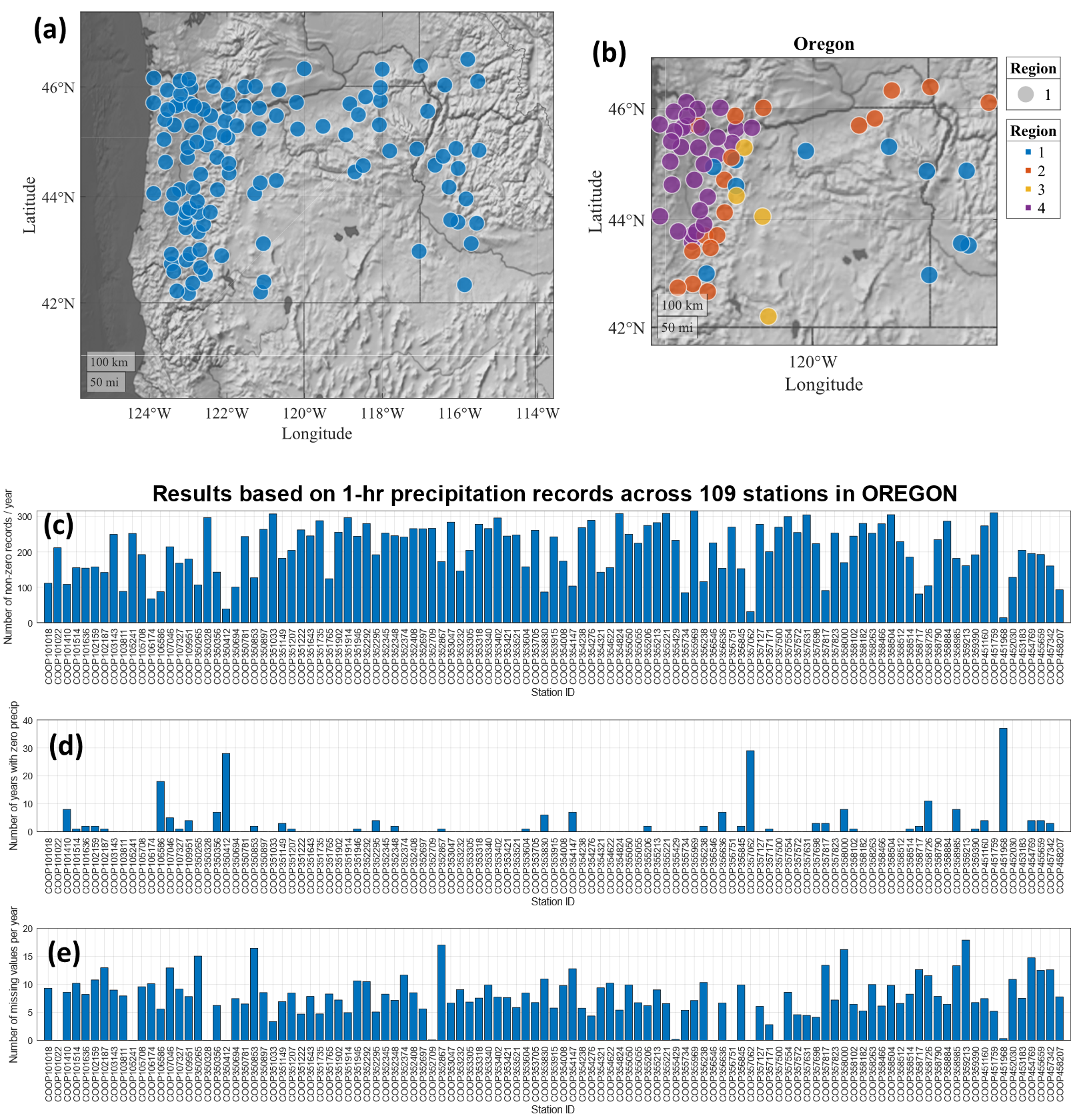
**

**Fig. S7** Spatial distribution of (a) 109 1-hr precipitation NOAA rain gauge stations selected within Oregon, (b) 63 NOAA rain gauge station selected for the NOAA-RFA in Oregon with the colors indicating the station locations selected as the homogeneous regions based on clustering and heterogeneity test, (c-d) bar plots showing the (c) number of non-zero records per year, (d) number of years with zero precipitation, and (e) number of missing values per year for the 109 NOAA rain gauge stations in Oregon.

**
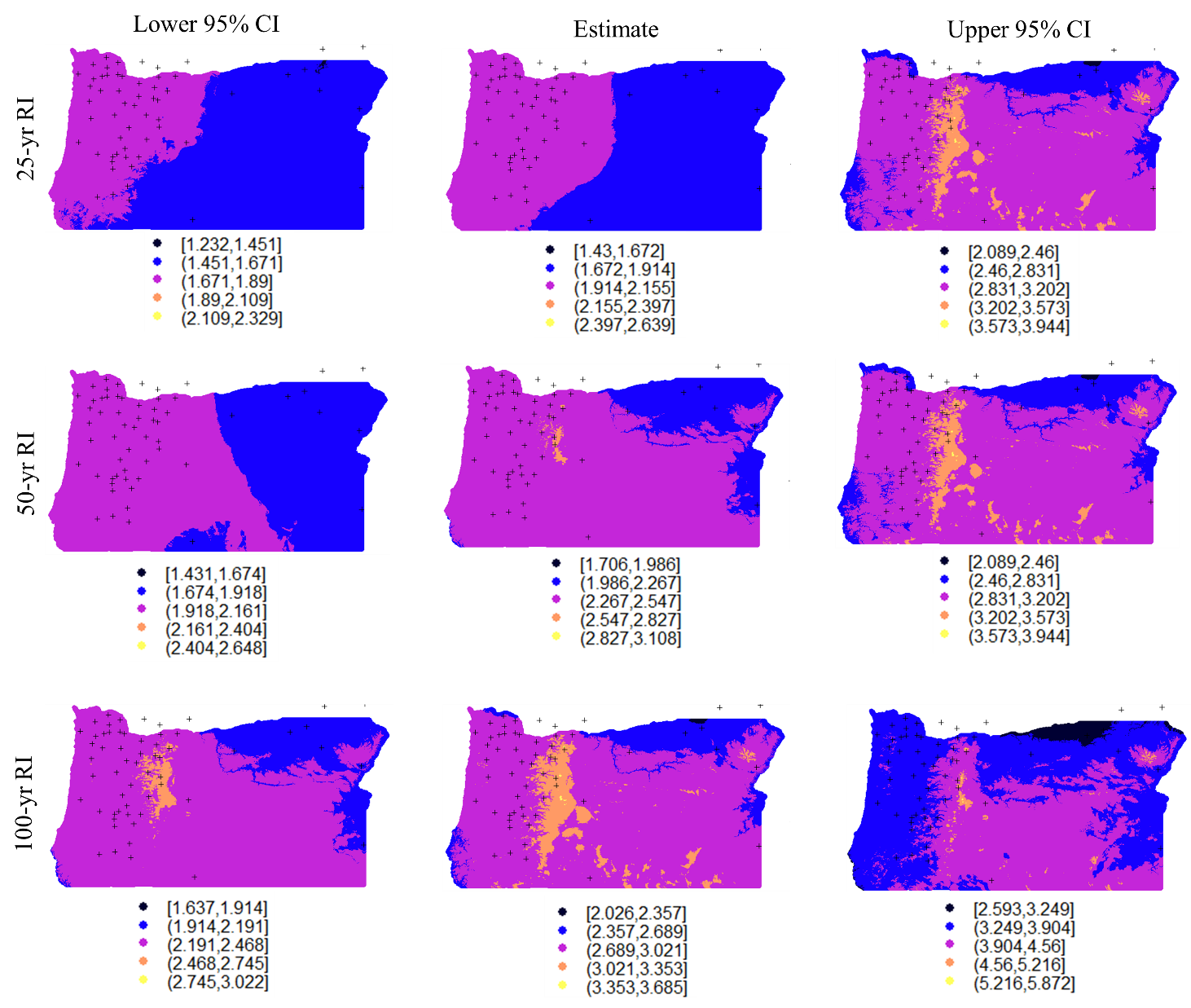
**

**Fig. S8** Geographical distribution of NOAA-RFA based PIs (cm/hr) for 25-yr, 50-yr, and 100-yr RIs for 1-hr duration storms obtained from spatial interpolation (by universal kriging) of NOAA station-based PIs in Oregon.

**
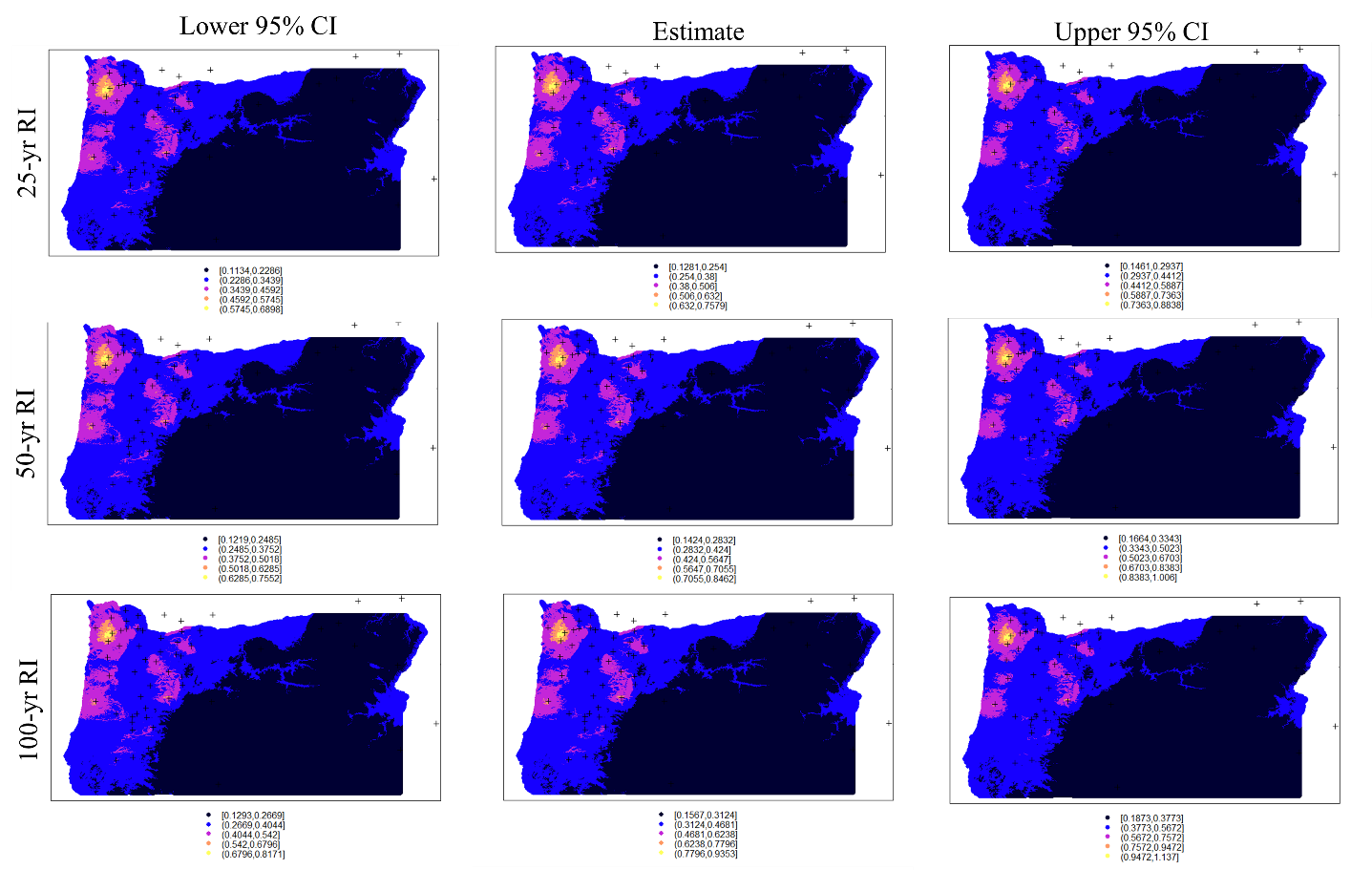
Fig. S9** Geographical distribution of NOAA-RFA based PIs (in cm/hr) for 25-yr, 50-yr, and 100-yr RIs for 24-hr duration storms obtained from spatial interpolation (by universal kriging) of NOAA station-based PIs in Oregon.


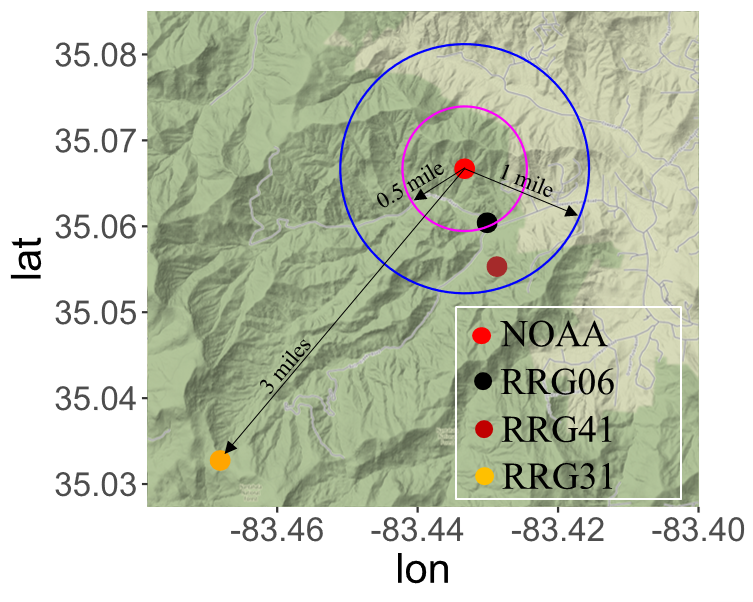


**Fig. S10** Spatial map showing the locations of NOAA gauge (COWEETA EXP STN) and onsite gauges, CHL-RRG06, RRG41, and RRG31 within the Coweeta Hydrological Lab EF.

**
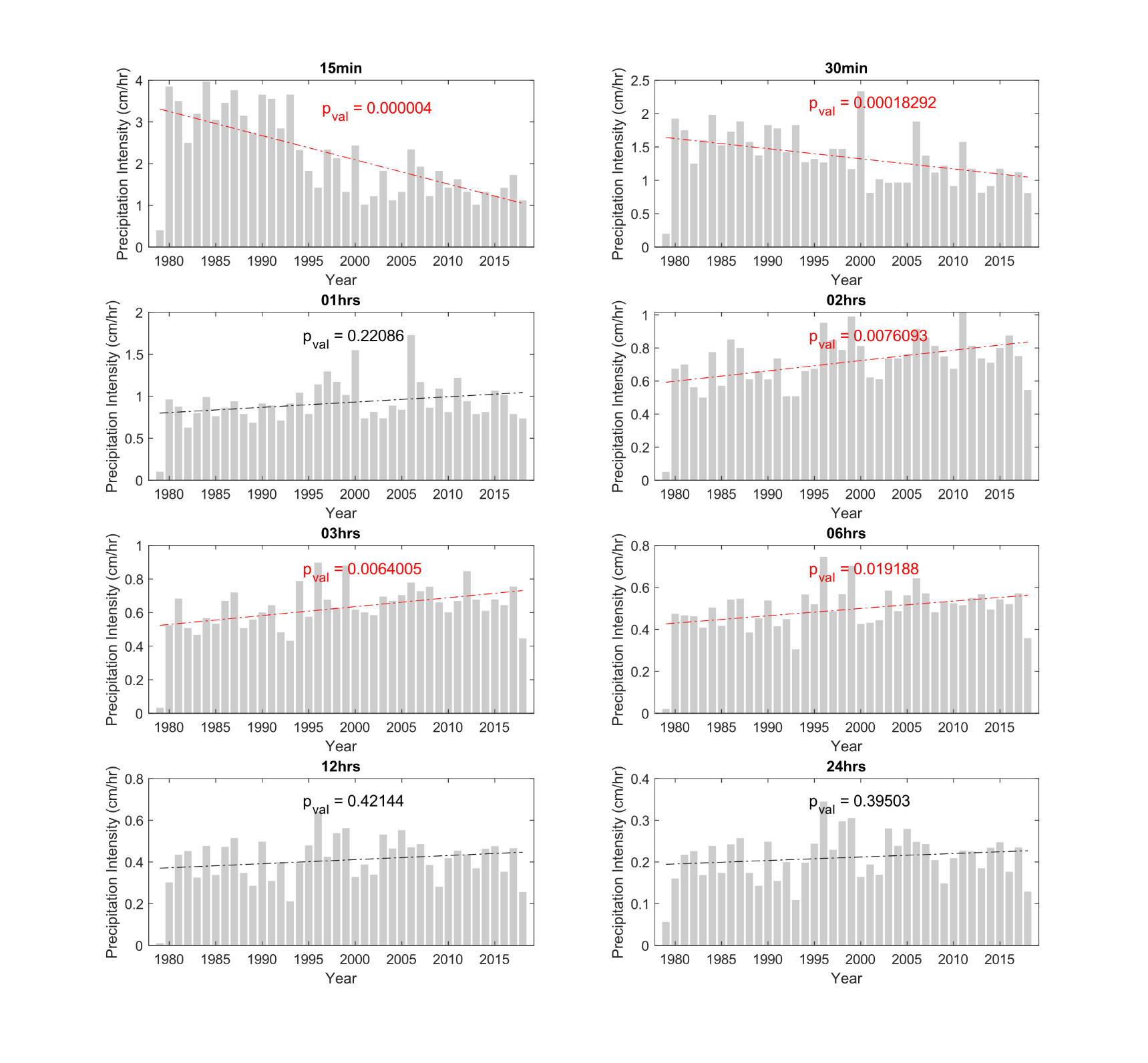
**

**Fig. S11** Time-series plot showing AMS of PIs and linear trends in the PIs at HJA-PRIMET station location. The p-values obtained from Mann-Kendall trend test (α = 0.05) are also shown, where a trend is considered statistically significant if p-value (shown in red) is found to be less than 0.05.

**
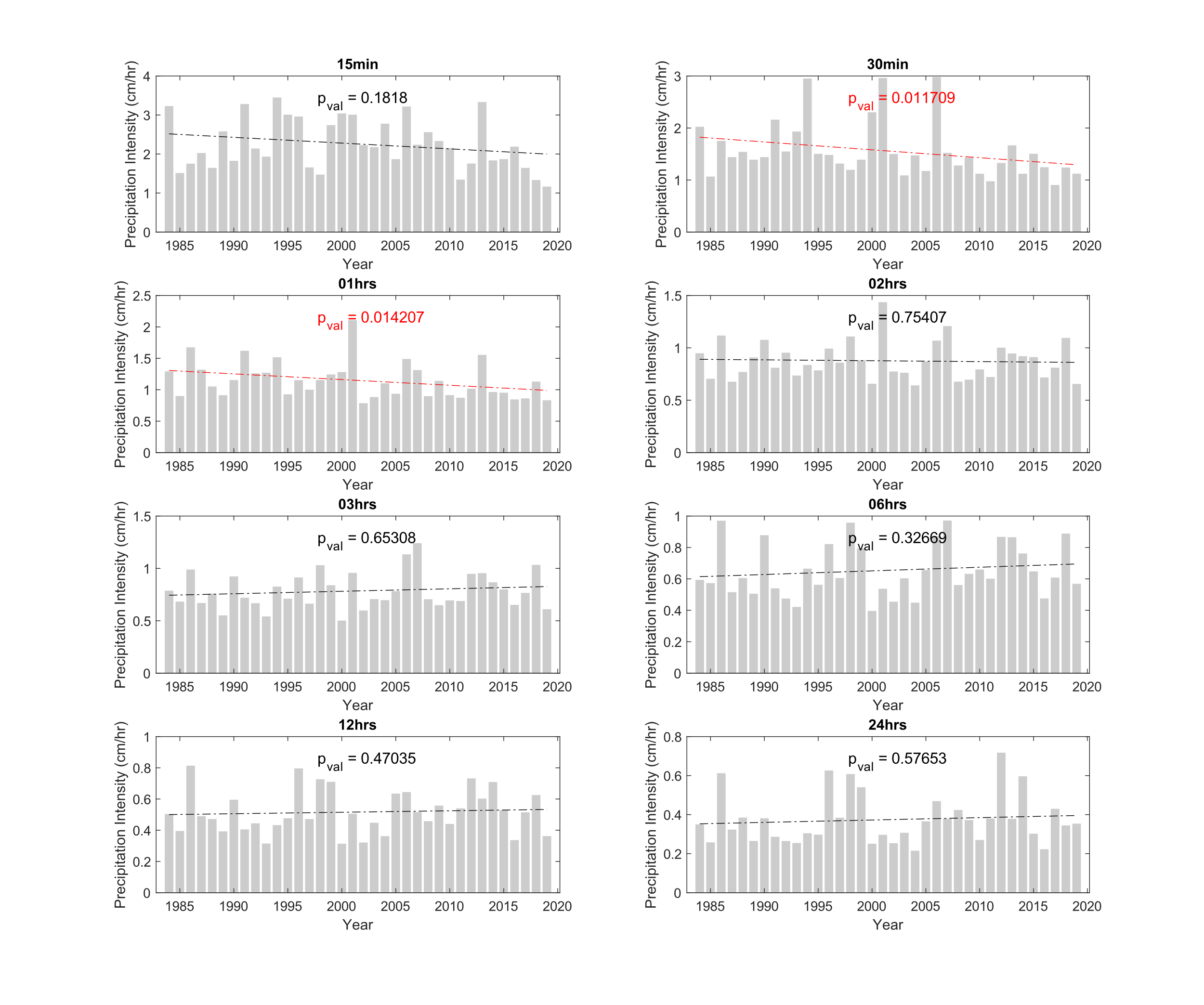
**

**Fig S12** Same as in Fig S5 but for the HJA-H15MET station location.

**
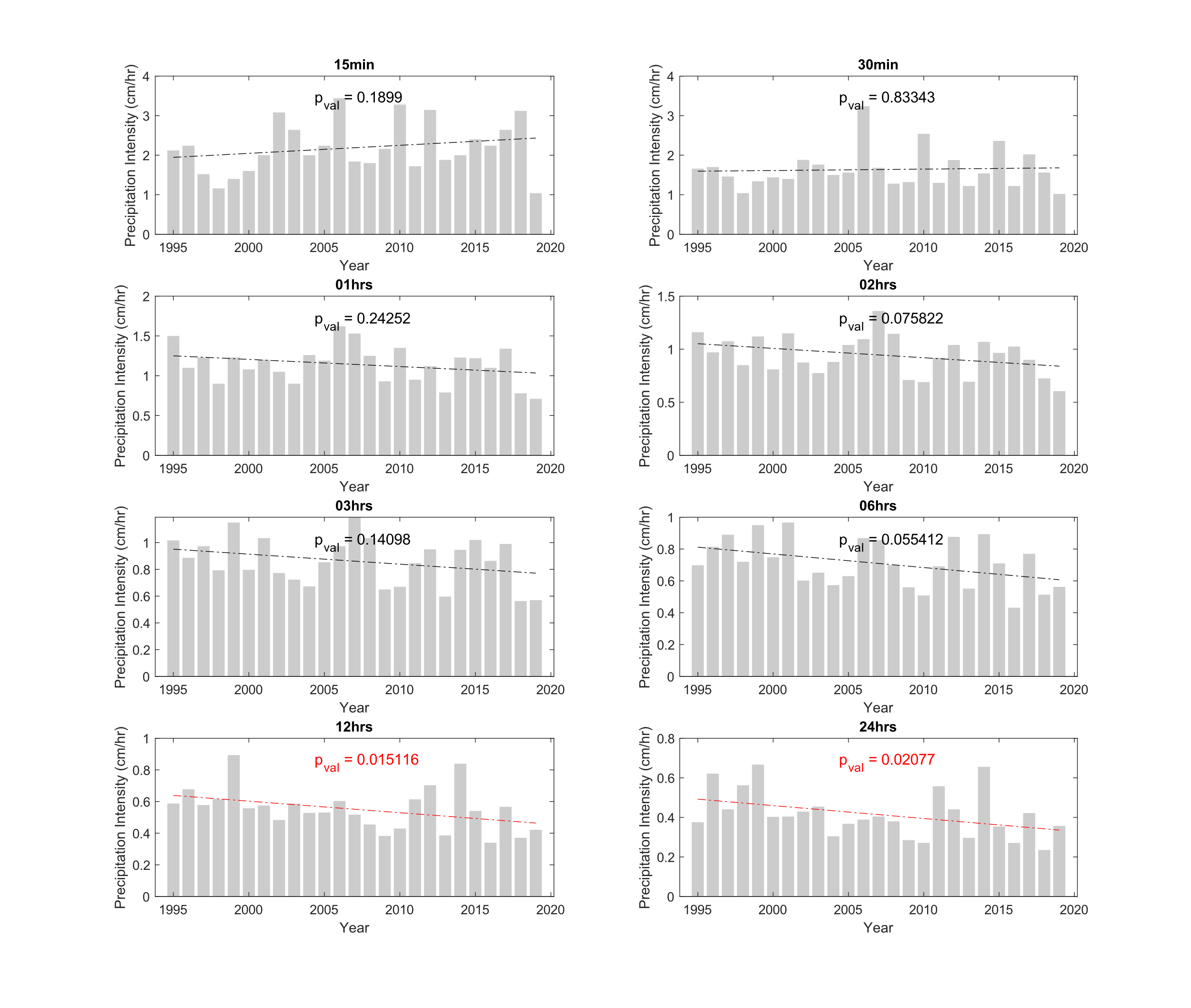
**

**Fig S13** Same as in Fig S5 but for the HJA-UPLMET station location.

**
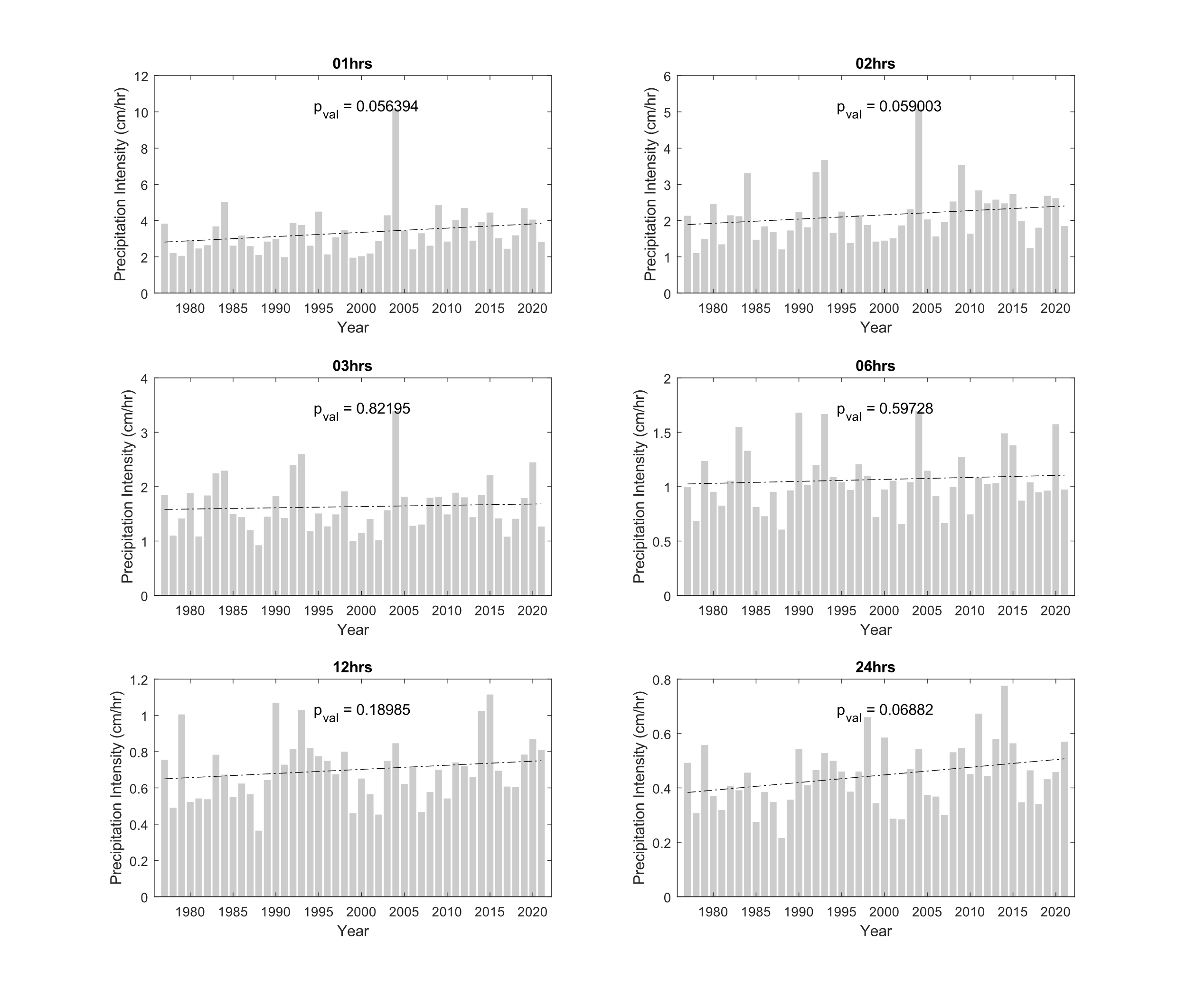
**

**Fig S14** Same as in Fig S5 but for the CHL-RRG06 station location.


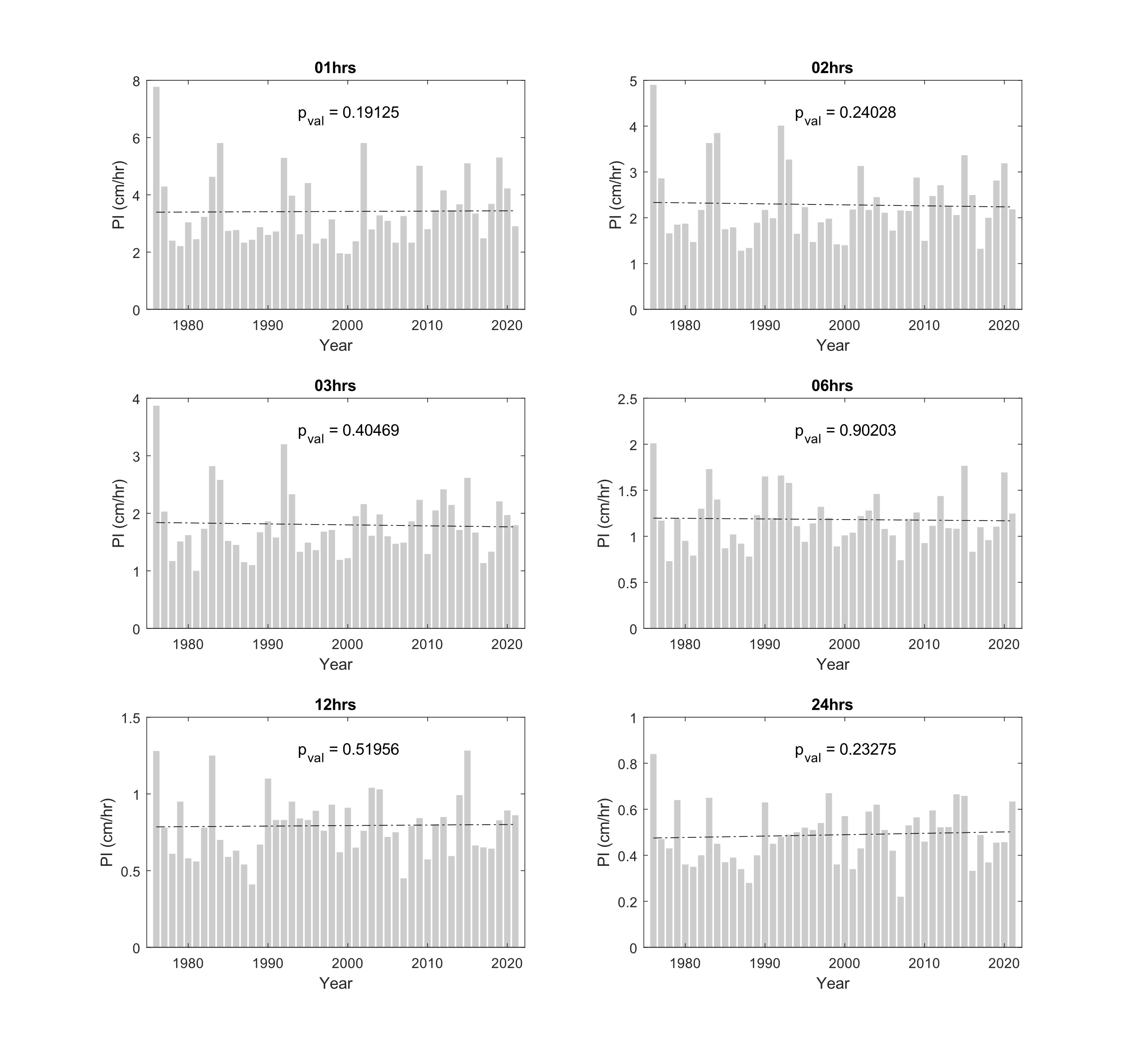


**Fig S15** Same as in Fig S5 but for the CHL-RRG41 station location.


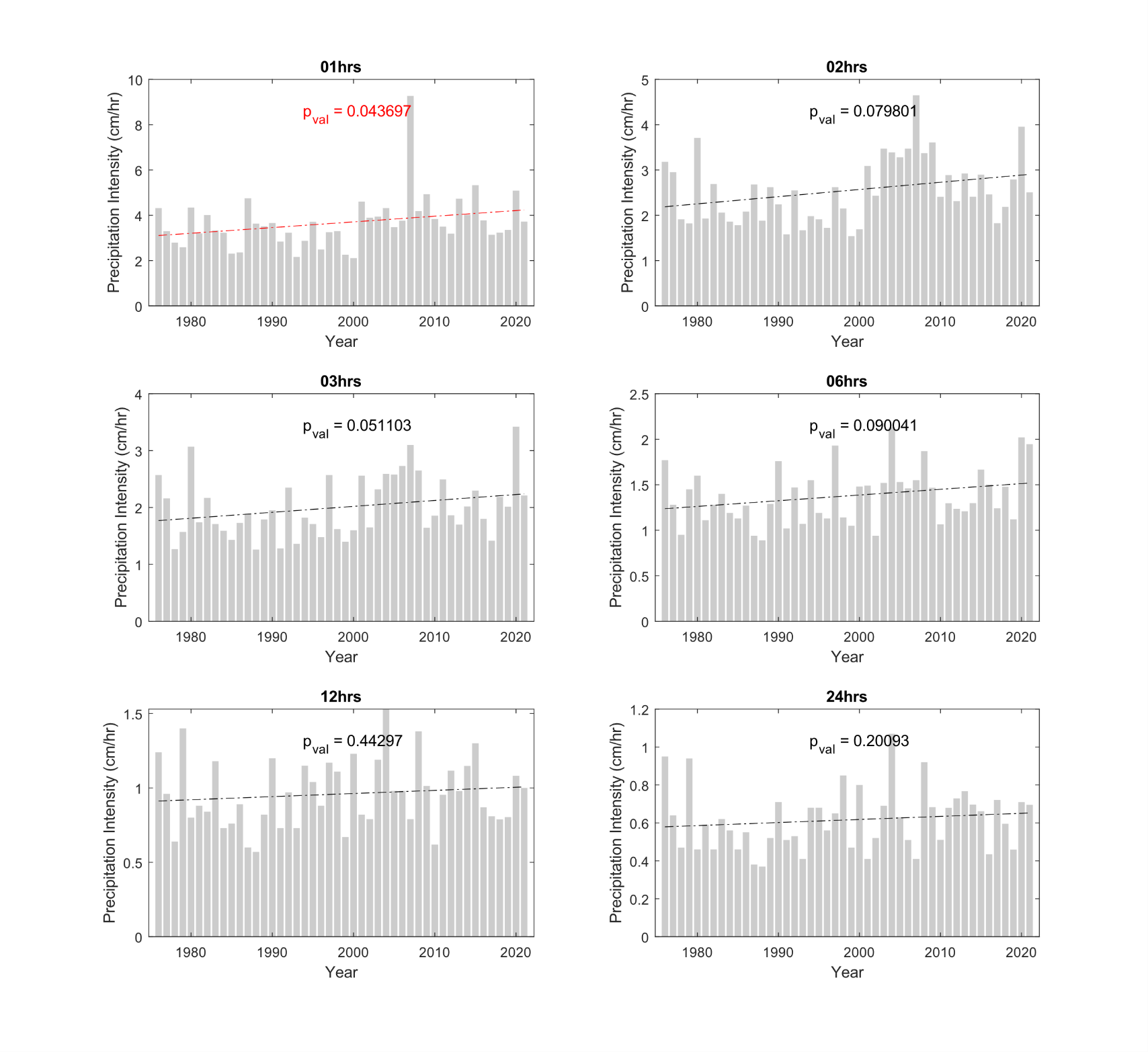


**Fig S16** Same as in Fig S5 but for the CHL-RRG31 station location.

**
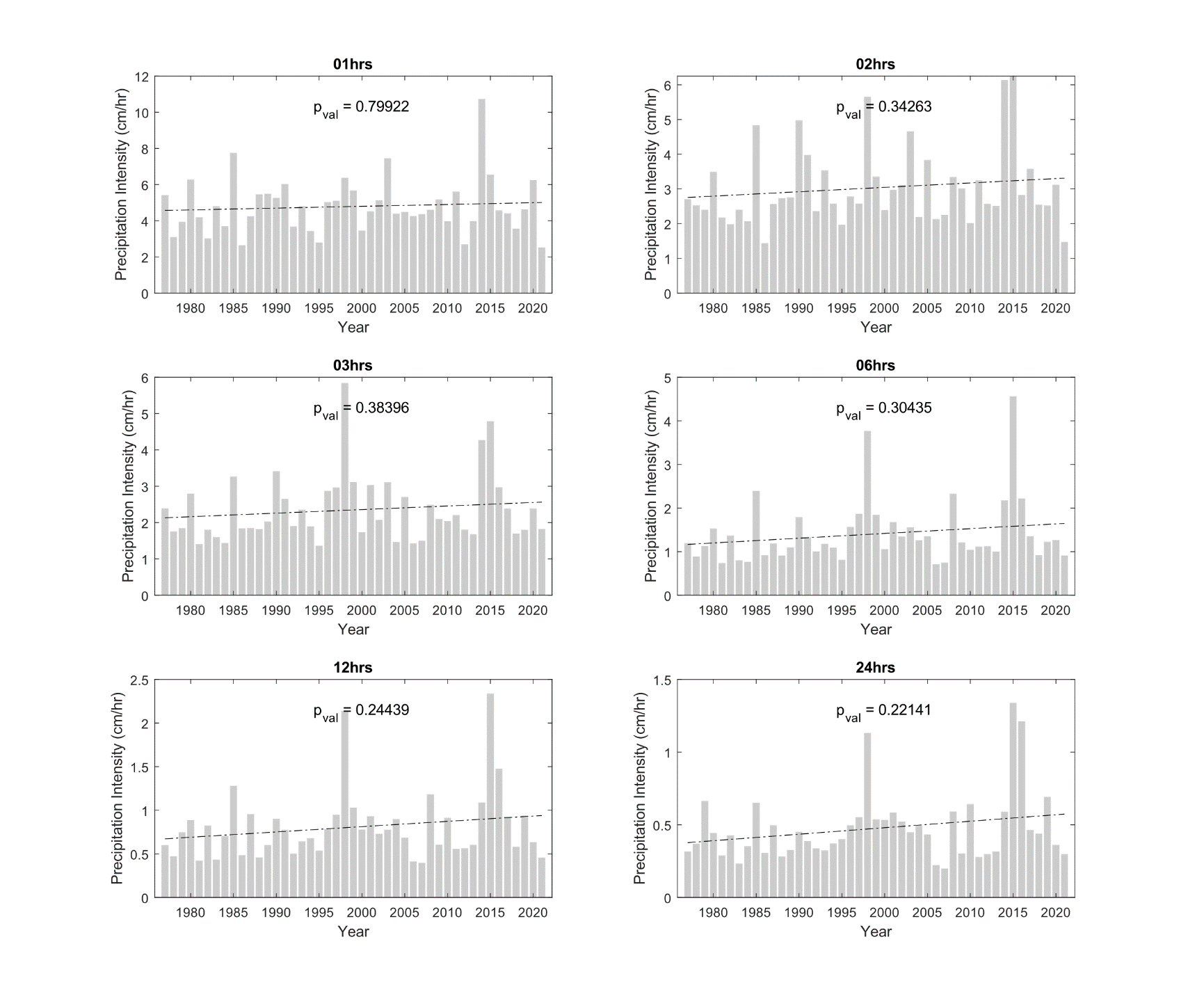
**

**Fig S17** Same as in Fig S5 but for the SAN-MET25 station location.

**
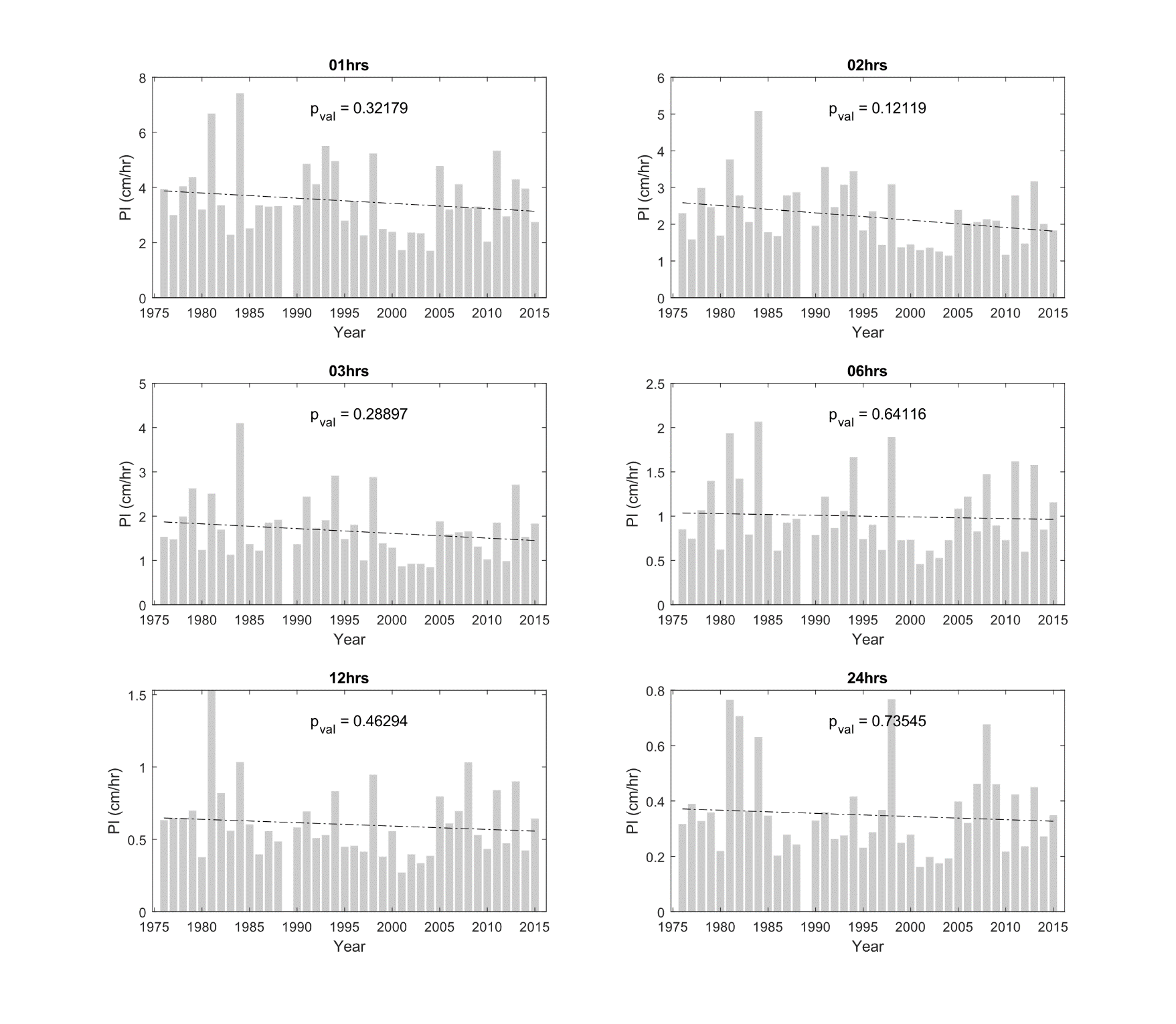
**

**Fig S18** Same as in Fig S5 but for the ALC-AC04 station location.

**
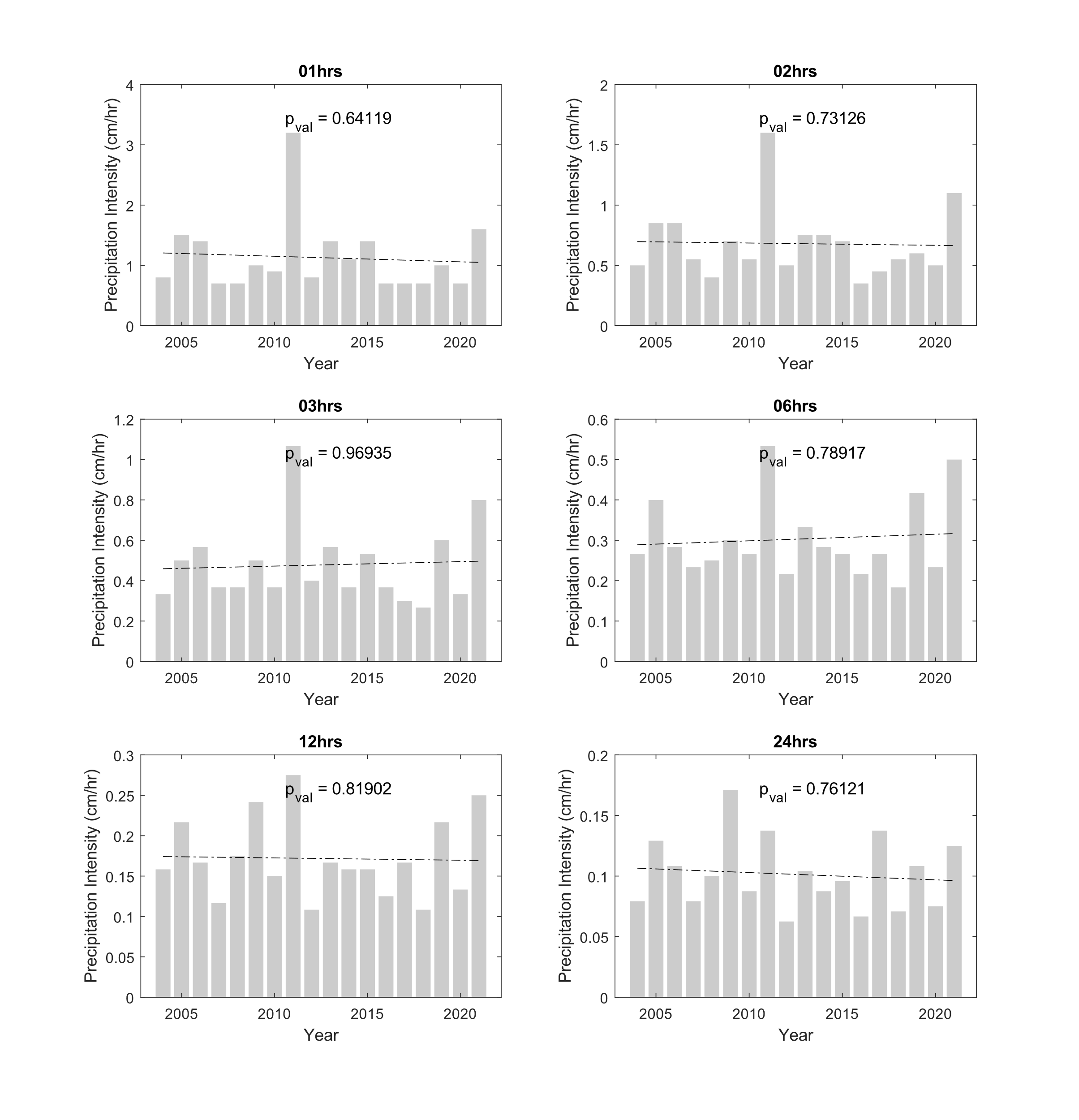
**

**Fig S19** Same as in Fig S5 but for the FRS-HQTRS station location.

**
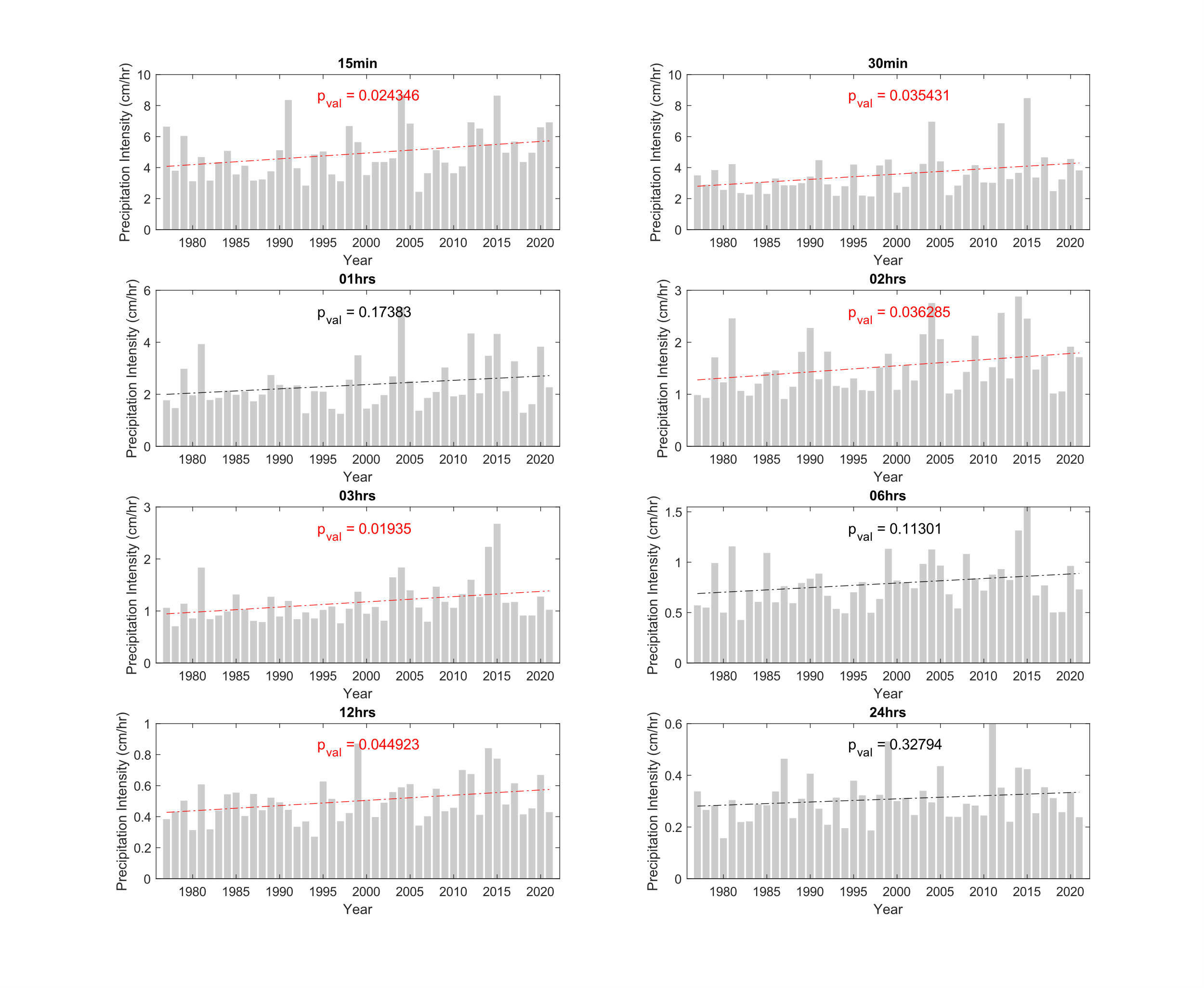
**

**Fig S20** Same as in Fig S5 but for the HBR-RG01 station location.

**References**

Bonnin, G., Martin, D., Lin, B., Parzybok, T., Yekta, M., Riley, D., 2006. Precipitation-Frequency Atlas of the United States. Volume 3 Version 4.0. Puerto Rico and the US Virgin Islands.

Hosking, J.R.M., Wallis, J.R., 1997. Regional frequency analysis.

Murtagh, F., Legendre, P., 2014. Ward’s Hierarchical Agglomerative Clustering Method: Which Algorithms Implement Ward’s Criterion? J Classif 31, 274–295. <https://doi.org/10.1007/s00357-014-9161-z>

Ngongondo, C.S., Xu, C.-Y., Tallaksen, L.M., Alemaw, B., Chirwa, T., 2011. Regional frequency analysis of rainfall extremes in Southern Malawi using the index rainfall and L-moments approaches. Stoch Environ Res Risk Assess 25, 939–955. <https://doi.org/10.1007/s00477-011-0480-x>

Srivastava, A., Grotjahn, R., Ullrich, P.A., Risser, M., 2019. A unified approach to evaluating precipitation frequency estimates with uncertainty quantification: Application to Florida and California watersheds. Journal of Hydrology 578, 124095. https://doi.org/10.1016/j.jhydrol.2019.124095

Yang, T., Shao, Q., Hao, Z.-C., Chen, X., Zhang, Z., Xu, C.-Y., Sun, L., 2010. Regional frequency analysis and spatio-temporal pattern characterization of rainfall extremes in the Pearl River Basin, China. Journal of Hydrology 380, 386–405. https://doi.org/10.1016/j.jhydrol.2009.11.013
